# Additive Manufacturing-Enabled Advanced Design and Process Strategies for Multi-Functional Lattice Structures

**DOI:** 10.3390/ma17143398

**Published:** 2024-07-09

**Authors:** Chinmai Bhat, Mayur Jiyalal Prajapati, Ajeet Kumar, Jeng-Ywan Jeng

**Affiliations:** 1 High-Value Biomaterials Research and Commercialization Center, National Taipei University of Technology, No. 1, Section 3, Zhongxiao East Road, Taipei 106, Taiwan; cvbhatjkd@mail.ntut.edu.tw; 2 Taiwan High Speed 3D Printing Research Center, National Taiwan University of Science and Technology, No. 43, Sec. 4, Keelung Rd, Taipei 106, Taiwan; mayurprajapati@mail.ntust.edu.tw; 3 Department of Mechanical Engineering, National Taiwan University of Science and Technology, No. 43, Sec. 4, Keelung Rd, Taipei 106, Taiwan; 4 Design for Additive Manufacturing & Innovation (DAMi) Lab, Department of Design, Indian Institute of Technology Guwahati, Guwahati 781039, Assam, India; 5 Academy of Innovative Semiconductor and Sustainable Manufacturing, National Cheng Kung University, No. 1, Dasyue Rd, East District, Tainan 701, Taiwan; 6 Department of Design, Indian Institute of Technology Guwahati, Guwahati 781039, Assam, India; 7 The Extreme Light Infrastructure (ELI ERIC), 252 41 Prague, Czech Republic

**Keywords:** lattice structures, lattice factors, additive manufacturing, multi-functional properties, design strategies, process strategies

## Abstract

The properties of each lattice structure are a function of four basic lattice factors, namely the morphology of the unit cell, its tessellation, relative density, and the material properties. The recent advancements in additive manufacturing (AM) have facilitated the easy manipulation of these factors to obtain desired functionalities. This review attempts to expound on several such strategies to manipulate these lattice factors. Several design-based grading strategies, such as functional grading, with respect to size and density manipulation, multi-morphology, and spatial arrangement strategies, have been discussed and their link to the natural occurrences are highlighted. Furthermore, special emphasis is given to the recently designed tessellation strategies to deliver multi-functional lattice responses. Each tessellation on its own acts as a novel material, thereby tuning the required properties. The subsequent section explores various material processing techniques with respect to multi-material AM to achieve multi-functional properties. The sequential combination of multiple materials generates novel properties that a single material cannot achieve. The last section explores the scope for combining the design and process strategies to obtain unique lattice structures capable of catering to advanced requirements. In addition, the future role of artificial intelligence and machine learning in developing function-specific lattice properties is highlighted.

## 1. Introduction

The field of additive manufacturing (AM) for multi-functional lattice structures is rapidly growing, with an increase in the demand for customization [1,2]. It integrates the concepts of 3D printing with the emerging design and process-based approaches to produce intricate, lightweight, and highly efficient components. Multi-functional lattice structures are engineered to fulfill multiple requirements concurrently. Some of the examples are lattice structures that are used in automotive and aerospace applications, with the intent of reducing weight without compromising the strength [3]. Conductive lattices are another example used to conduct heat and electricity along with providing the required structural integrity to the components [4]. Furthermore, lattice structures are also used as heat exchangers by providing a sufficient surface-to-area ratio along with providing mechanical strength against a load [5,6].

Lattice structures are formed by the strategic placement of the basic functional blocks known as unit cells. The unit cells are characterized by the size and arrangement of their individual/surface/plate components, which are joined/connected at specified nodes/surface/edges [7,8,9]. These lattice structures exhibit characteristics of both a structure and a material. At the level of the unit cell, this can be regarded as a structure in terms of its characteristics and properties. However, when it is tessellated in the design space and its homogenized characteristics are assessed on a larger scale, it behaves like a material [10]. 

By adjusting the parameters, such as cell topology (connectivity) or cell geometry (unit-cell size, truss, or plate dimensions), it is possible to greatly modify the physical properties, such as acoustic, dielectric, and mechanical. These modifications cannot be achieved using original materials alone [11]. The four main factors that influence the characteristics of lattice structures are: (a) The properties of the material, (b) The cell topology and cell geometry of the unit-cell cellular structure, (c) The relative density of the lattice structure, and (d) tessellation of unit cells for the design space [12,13].

This review focuses on classifying various methodologies adopted to manipulate the lattice structures’ basic factors: properties of the material, cell topology/morphology, relative density, and tessellations. This study compiles all the research carried out to date to manipulate these four basic factors. Through these manipulations, tunable functional and structural properties can be achieved for customization. This review paper classified all these four factors into either design-based or process-based approaches. The manipulation of morphology (i.e., multi-morphology), relative density (i.e., functional grading), and tessellations is considered a design-based approach. On the contrary, based on the material properties, tunable functionalities can be obtained with the help of multi-material approaches. These multi-material lattice structures can be obtained through the manipulation of processes. The authors believe that comprehensive knowledge about the basic factors and their manipulation for customization would greatly benefit the upcoming research on AM fabricated lattice structures. Such a study has not been carried out to date, and thus, this review paper would contribute greatly to the AM community. The subsequent sections of the manuscript are framed as follows:

Section 2 elaborately explains various design strategies adopted to obtain the multi-functional lattice structures. These include the strategies of functional grading, multi-morphology, and tessellation. The section briefly explains the role of nature in developing such strategies and highlights its salient features and applications.

Section 3 illustrates various process-based approaches to obtain multi-material lattice structures that can serve multi-functional purposes. Various types of composites, such as fiber-reinforced composites, particle-reinforced composites, and nanocomposites that are fabricated through additive manufacturing processes, are explained in detail. Furthermore, the section also explains the novel closed-cell lattice structures with secondary filling strategies to avail the properties of both the closed-cell and filled materials.

Section 4 provides various recommendations for future studies that can be carried out to obtain multi-functional lattice structures. Various AI/ML tools can be utilized to enhance the efficacy of the design and process strategies that are included in this study to provide excellent function-specific properties. The future outlook is followed by the Conclusions Section (Section 5).

## 2. Design Strategies to Obtain Multi-Functional Properties

### 2.1. Functionally Graded Lattice Structures

The grading of the cellular lattice structures based on functional necessity is known as ‘Functional grading’ [14]. Functional grading is particularly important when mechanical, physical, and/or geometrical properties are required to be tailored spatially in order to meet biological, mechanical, and/or thermal requirements concurrently [15,16]. Liu et al. classified the functional grading phenomenon into compositional, micro-structural, and geometric factors [17]. Functional gradients are essentially featured by the creation of site-specific properties distributed within a material that originates from variations in factors, such as composition, micro-structure, and geometry. Compositional and micro-structural grading are quite challenging due to metallurgical and fabrication limitations. However, with the advent of additive manufacturing, achieving functional grading through structural/geometrical manipulation is possible to obtain different properties at different depths.

#### 2.1.1. Classification of Functionally Graded Lattice Structures

**Figure 1:**
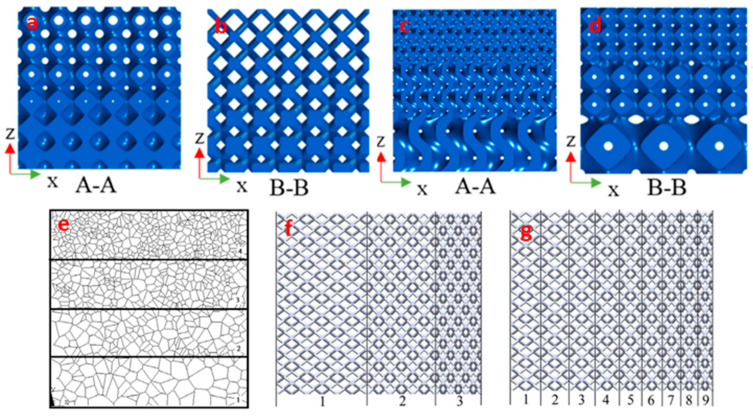
Various functional grading strategies being employed to obtain desired mechanical and functional properties: (**a**) surface-based thickness grading, (**b**) strut-based thickness grading, (**c**,**d**) surface-based unit-cell size grading, (**e**) stochastic grading, (**f**) step-wise grading, and (**g**) continuous grading.

Functionally graded lattice structures are categorized into thickness grading and size grading based on the design strategy involved [18]. In thickness grading, the relative density of successive layers of lattice structure increases by a stipulated amount. This is achieved by maintaining a uniform basic unit-cell size and manipulating the shell thickness, as shown in Figure 1a,b. On the contrary, the basic unit-cell size is varied and shell thickness is maintained constant in size grading (Figure 1c,d) [19,20]. Another less common grading strategy involves stochastic grading by manipulating the stochastic ratio in each step, as shown in Figure 1e. Functional grading strategies can also be classified into step-wise and continuous grading, as depicted in Figure 1f,g, respectively [21].

#### 2.1.2. Inspiration from Nature

**Figure 2:**
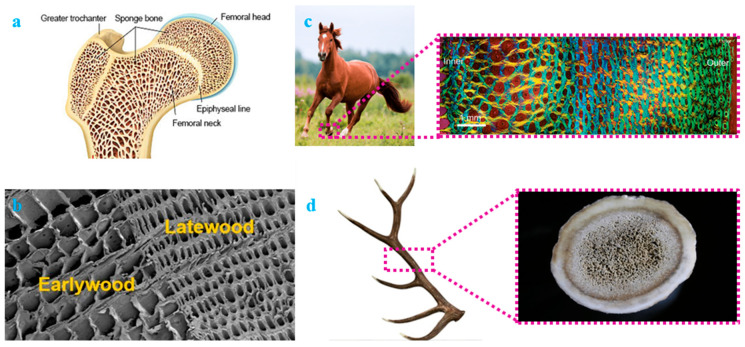
Concept of functional grading in nature: (**a**) trabecular bone, (**b**) earlywood and latewood of Norway spruce, (**c**) cross-section of Equine hoof, and (**d**) cross-section of Elk antler.

Nature is the best place to study the concept of optimization as billions of years of evolution create adaptation-based optimized designs. The functional grading concept evolves from this natural adaptation of many creatures, where the demand is to fulfill various functional requirements in the most optimized way. Several fish scales, such as Senegalus, Spatula, Gigas, etc., adopt the concept of functional grading to obtain stiffer and harder external regions with a softer internal base [22,23,24]. Trabecular bone is one of the best examples of functional grading, with an outer stiff portion due to less porosity/more material and a flexible inner portion due to excessive porosity in the core. A cross-section of the trabecular bone is shown in Figure 2a. Similarly, the earlywood and latewood of Norway spruce show a functional grading strategy (Figure 2b) [25,26]. Earlywood, being more porous, helps in the supply of necessary elements from the roots to the tip of the tree. On the other hand, latewood protects the stem against harsh environmental conditions. The cross-section of an Equine hoof (Figure 2c) also displays function grading with more material toward the outer direction to have excellent mobility against a harsh and rugged terrain [8,27]. Figure 2d shows an Elk antler, which has a functional grading porosity distribution with a softer and porous core and hard covering [28,29,30]. The high-strength outer covering helps the antler protect itself from various adversaries. A similar strategy was adopted by many researchers in various structural and functional applications. With the advent of additive manufacturing, the design and fabrication of these structures are possible with ease [31,32].

**Figure 3:**
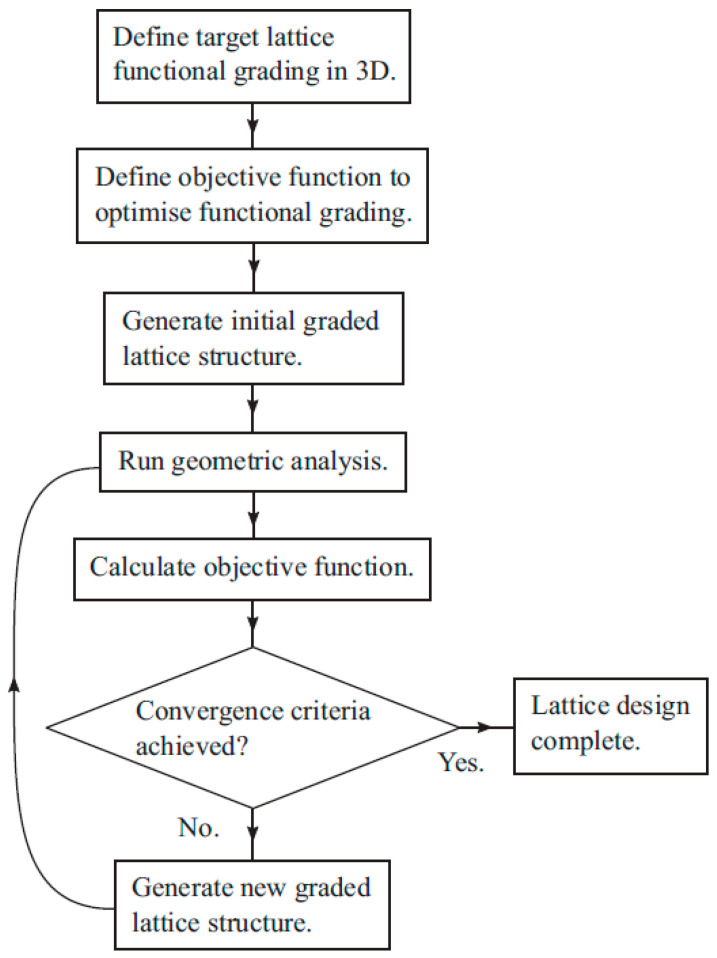
Proposed general approach to functionally graded lattice structure design [33].

Maskery et al. proposed the algorithm shown in Figure 3 to predict the usability of functional grading based on finite element analysis [33]. In designing a functionally graded lattice structure, defining the functional objectives are an important step. Once the objective functions are set, the functional grading design is generated by modifying the parameters such as relative density and unit-cell size based on the obtained feedback from the algorithm [33].

#### 2.1.3. Applications of Functionally Graded Lattice Structures

(a) Energy absorption

**Figure 4:**
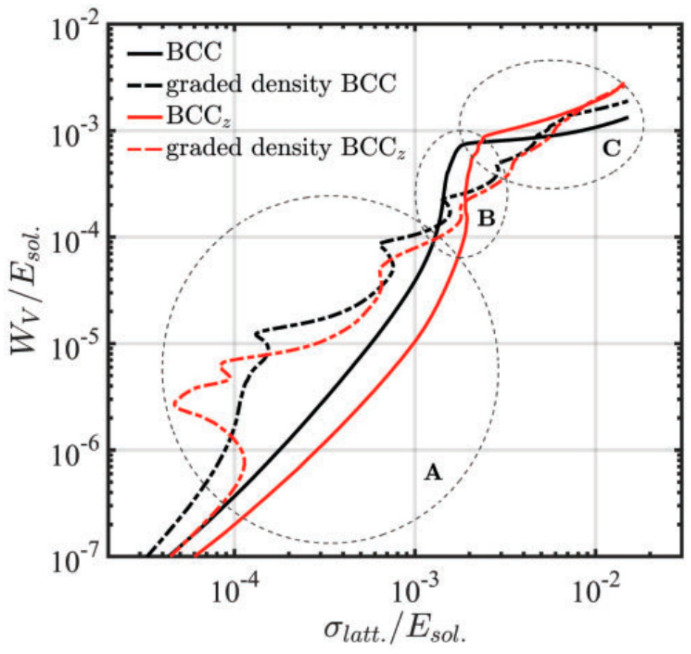
Comparison of normalized energy absorption capacity of functionally graded and non-graded lattice structures (BCC and BCCz) [34].

Several researchers have exploited the concept of thickness grading to improve the energy absorption of the lattice structures [20,34,35,36,37,38,39]. Al-saedi et al. designed a thickness-graded F2BCC strut-based lattice structure to obtain enhanced specific energy absorption [35]. A similar study was also carried out by Maskery et al. with BCC and BCCz strut-based lattice structures [34]. Figure 4 shows the normalized energy absorption of graded and non-graded lattice structures [34]. The Y-axis in Figure 4 represents cumulative energy absorption (Wv) against stress (in the X-axis), both of which are normalized with the elastic modulus of the lattice structure. On the other hand, the X-axis in Figure 4 regions A, B, and C represent the elastic, plateau, and densification regions of non-graded structures, respectively. The collapse of the first two layers of thickness-graded structures is also included in region A. It can be observed in Figure 4 that thickness-graded structures absorb more energy than non-graded structures in region A [34].

**Figure 5:**
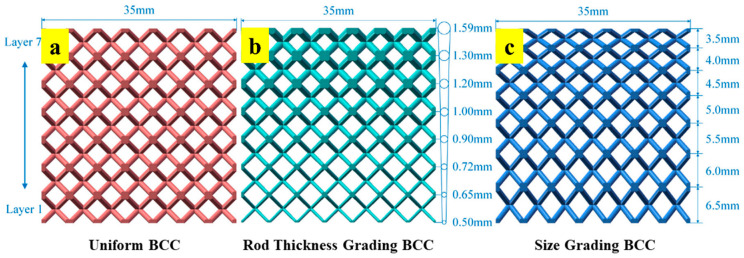
Comparison of BCC strut lattice structure: (**a**) uniform, (**b**) thickness grading, and (**c**) size grading [37].

Bai et al. modified the uniform-density BCC strut-based lattice structure (Figure 5a) into thickness-graded (Figure 5b) and size-graded (Figure 5c) structures, and compared their energy absorption capacities. The study concluded that the energy absorption of size-graded structures was higher than that of thickness grading and uniform lattice structures. This was attributed to the non-linear nature of the curve in size-graded structures, which covered a greater area under the curve compared to the linear nature of the uniform structure. Furthermore, the greater densification strain in the graded structure compared to uniform structure also contributed to increased energy absorption [37]. The performance of thickness grading was found to be inferior due to the excessive thinning of strut diameters [37]. A similar study was also conducted by Plocher et al. and concluded with some important findings. The study concluded that the density grading of structures enhances the mechanical and functional properties of lattice structures up to a certain optimized grading strategy. Excessive grading makes some portions of the lattice structure ineffective against load, thereby reducing the performance of the lattice structure [20]. Zhao et al. studied the energy absorption capacity of surface-based TPMS (triply periodic minimal surface) structures and found out that energy absorption was increased by 60% by adopting a density grading strategy [38].

(b) Fail-safe designing

Apart from energy absorption, some research is available presenting enhanced plateau stress using a density grading strategy [39,40]. Flattened or incremental plateau stress is considered a fail-safe design compared to decremental plateau stress. This is due to the fact that lattice structures with a decremental plateau stress tend to lose strength post-yielding, leading to a catastrophic failure [41]. Choy et al. evaluated the plateau stress and energy absorption capacity of graded-density cubic and honeycomb strut lattices and compared them with uniform-density structures. It was found that the plateau stress and energy absorption of density-graded lattice structures were 67% and 72% more than uniform lattice structures, respectively [39]. Brothers et al. obtained a uniformly rising plateau stress (i.e., incremental plateau stress) by density grading, which is advantageous over uniform-density structures that exhibit a uniform plateau stress [40]. The increasing plateau stress is considered to be a fail-safe design. The yield strength and elastic modulus of the structures were also observed to increase with increasing the extent of density grading [42].

(c) Impact and blast resistance

**Figure 6:**
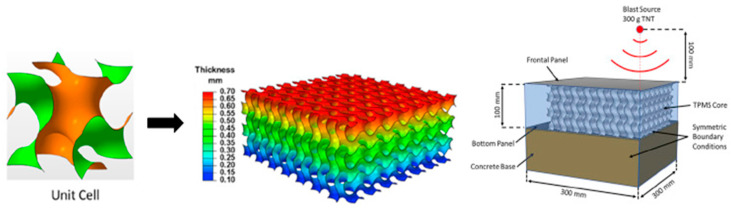
Functionally graded TPMS Gyroid lattice structure composite for blast-resistance applications [47].

Density grading is also known for providing an excellent dynamic response under impact loading conditions. Zeng et al. found that energy absorption was observed to be improved with density grading under impact loading [43]. Hence, density grading proves to be an important strategy that can be exploited for lightweight blast-resisting structures. Several studies are available, focusing on functional grading strategies being employed in 2D sandwich structures to achieve better resistance against high-velocity impact. This property is achieved by adding more density to the surface that takes the high-velocity impact loading, thus absorbing maximum energy upon initial contact. Subsequent energies were absorbed in steps with the lower density of structures [43,44,45,46]. Figure 6 shows a TPMS Gyroid lattice structure, which is functionally graded to study the blast-resistance capacity of the structure. The blast source of 300 g of TNT was applied to this sandwiched structure. The study focused on evaluating specific energy absorption and force transfer to the concrete foundation. The effectiveness of density grading in blast resistance was revealed with less force transmission to the bottom concrete plate compared to the uniform-density Gyroid structure. Moreover, plastic deformation was observed to be uniform in the case of functionally graded lattice against unstable plastic deformation in uniform-density lattice structure [47].

**Figure 7:**
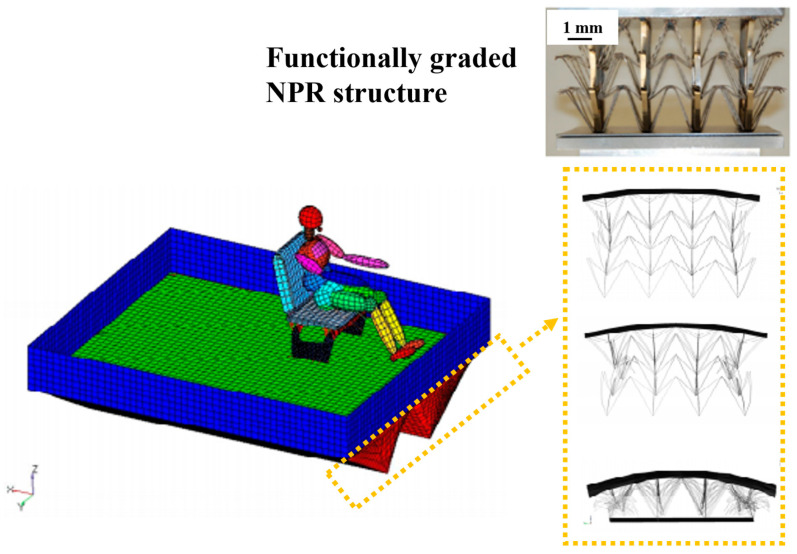
Functionally graded negative Poisson’s ratio (NPR) lattice deflector for protection against crash or impact [48].

Similarly, Ma et al. proposed functionally graded negative Poisson’s ratio structures depicted in Figure 7 for a blast protective deflector. The designed structures adaptively concentrate more material into areas where maximum stress is induced, thereby mitigating the possibility of damage by crash [48].

(d) Manipulation of failure patterns

**Figure 8:**
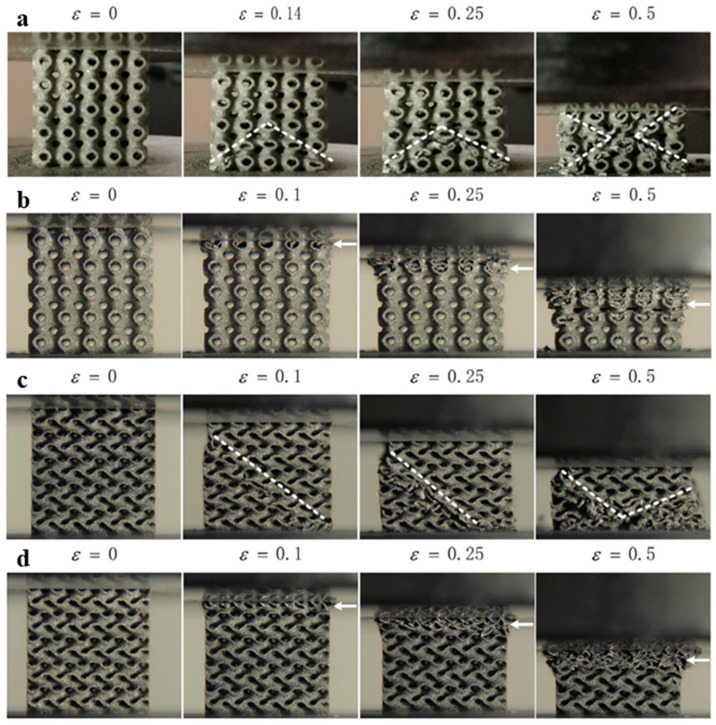
Failure patterns of different lattice structures: (**a**) shear failure of uniform Schwarz-P lattice structure, (**b**) layer-wise failure of functionally graded lattice structure, (**c**) shear failure of uniform Gyroid lattice structure, and (**d**) layer-wise failure of functionally graded Gyroid lattice structure [38].

The density grading strategy was also exploited to manipulate the pattern of failure of the lattice structure [20,35,37,38,39]. Some uniform-density lattice structures fail catastrophically with single- or double-shear band failure. Such failures are not considered a fail-safe design. Adopting a density grading strategy in such structures helps convert the failure of lattice structures to layer-wise failure, which is gradual and fail-safe compared to shear failure. Zhao et al. studied the failure pattern of the lattice structures as shown in Figure 8. Figure 8 shows ungraded Schwarz-p and Gyroid structures exhibiting double-shear band failure and single-shear band failures, respectively. On the other hand, adopting the strategy as per Figure 8d, the grading strategy converts the shear failure of these TPMS structures to layer-wise failure, thereby making the structural design a fail-safe one [38].

(e) Functionally graded lattice composites

**Figure 9:**
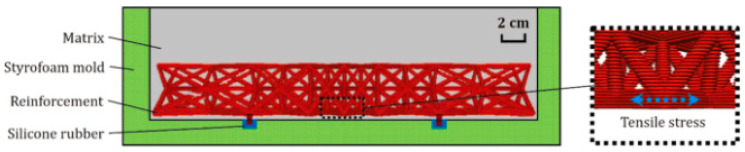
Three-dimensional-printed ABS reinforced functionally graded lattice structure in cementitious material [49].

Xu et al. prepared cementitious composites with functional graded polymeric lattice structures made up of ABS material [49]. The study aimed to provide alternate reinforcement material, other than steel, which is corrosive and expensive. Figure 9 shows samples prepared with functionally graded ABS reinforcement with cementitious material as the matrix. Samples prepared with the reinforcement of ABS lattice structures showed better ductility than plain cementitious material, and the nature of cracking was converted from brittle to ductile failure. Hence, 3D-printed polymeric lattice structures can be used as an alternative to steel for reinforcement. The study also highlighted the advantages of using density grading in modifying the flexure performance of reinforced cementitious composites. With optimized density grading, 47.48% of the reinforcement material was reduced with a 57.18% increase in ductility failure strength [49].

### 2.2. Multi-Morphology Lattice Structures

Each basic unit cell has its own structural and functional response that is compiled and reflected in the lattice structures. Based on the different responses, the lattice structures are classified into bending- and stretch-dominated behaviors. Even in the same domain of behavior, different unit cells provide different structural and functional responses, such as stiffness, peak strength, energy absorption, and densification strain. For instance, both FCC and octet trusses belong to the same category of stretch-dominated behavior. Yet, the FCC truss showcases a better peak strength and elastic modulus, whereas the octet truss demonstrates better energy absorption. Designing a lattice structure with multiple combinations of these basic unit cells would create tuned deformation characteristics (i.e., multi-functionality can be achieved). Heterogeneity can be engineered by spatially distributing regions of different unit-cell architectures to create multi-morphology lattices. The term ‘multi-morphology’ is sometimes also called ‘hybrid lattice structures’. The major aim of designing multi-morphology lattice structures is to take advantage of the inherent mechanical performance of each type of unit cell. Unlike composites composed of traditional materials, these multi-morphology lattices can harness multiple functionalities, despite being composed of a single base material.

**Figure 10:**
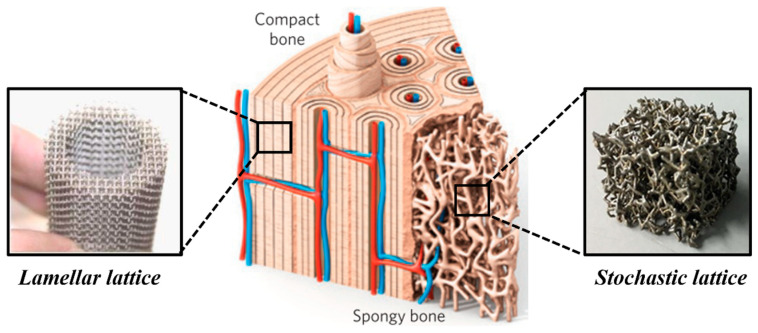
Multi-morphology strategy inspired by bone, where spongy bone is replicated by a cushioning, bending dominated stochastic structure, and compact bone is replicated by a load-bearing stretch-dominated lamellar structure.

The concept of multi-morphology lattice structures has recently begun to receive attention through a few exemplary studies [14,16,19,26,27,50,51]. The inspiration can be traced back to nature, where an abundance of such structures exists based on adaptation. One such example is the macroscopic arrangement of bone [52]. The macroscopic arrangement of bone involves compact/cortisol bone at the surface and spongy/trabecular bone in the interior, as shown in Figure 10. The role of the cortisol bone is to provide strength and housing to osteons and the haversian canal, which cover blood vessels, whereas the role of spongy bone is to accommodate sudden shocks and cushion effects [52].

Furthermore, the domain delivers design flexibility in terms of controlling the unit-cell size, the unit-cell type, and the unit-cell porosity. Such design freedom provides an avenue to better tailor the structure to meet the engineering requirements.

#### 2.2.1. Surface-Based Multi-Morphology Lattice Structures

One of the major challenges associated with the design of surface based multi-morphology lattice structures is their inability to blend in with the different morphologies. A poor interface may result in premature lattice failure, which would hinder us from realizing the true potential of these structures. Over the years, researchers have proposed several methodologies and tools to minimize interface heterogeneity. Most of them have used TPMS-based unit-cell morphologies for their studies [15,16,33,53]. The ease of manipulating and combining implicit surface functions associated with these structures makes it a more feasible option to create one cell type transiting into another [33].

Rastegarzadeh et al. have reported the design frameworks using the artificial neural network to smoothen the sharp transition between two different morphologies by using a the linear interpolation operation [54]. Such a method would help reduce the stress concentration, thereby increasing the durability of the structures [54]. Yoo and Kim proposed the design algorithm to smoothly bridge two TPMS morphologies [26]. The ‘*sigmoid function (SF)*’, depicted as Equation (1), is linked with the ‘k’ factor, which would determine the transition between two morphologies [16,26].

(1) $$\mu \left(x,y,z\right)=\frac{1}{1+e^{-kG\left(x,y,z\right)}}$$

where *G*(*x*,*y*,*z*) = 0 is the transition boundary between two TPMS pore morphologies, and *k* is a constant that determines the transition gradient. That is, a large value of *k* results in a more abrupt transition between two pore morphologies [16,26,50]. By evaluating the sigmoid function, the equation of the whole multi-morphology can be described as shown in Equation (2) [26,50]:

(2) $$\varphi h\left(\mu \right)=\left(1-\mu \right)\varphi 1+\mu \varphi 2$$

where *φh*, *φ*1, and *φ*2 represent the functions of hybrid pore morphology and the parent morphologies 1 and 2, respectively. ‘*µ*’ is a blending parameter that satisfies *µ*(*x*,*y*,*z*) ∈ [0, 1].

**Figure 11:**
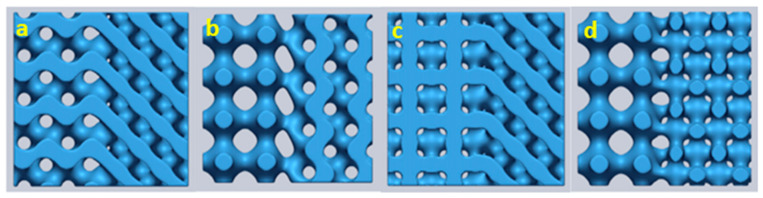
Multi-morphology TPMS structures with a sigmoidal function for smooth transition from one morphology to another: (**a**) Gyroid Diamond, (**b**) Primitive Gyroid, (**c**) IWP Diamond, and (**d**) Primitive IWP *(Note: IWP is abbreviated as I-graph and wrapped-package graph)* [16].

Figure 11 shows multi-morphology lattice structures comprised of two different TPMS unit cells. The hybridized structures shown in Figure 11 are derived using Equation (2) [26]. It can be observed that the transition from one morphology to another is rather smooth and gradual. Apart from the sigmoidal function shown in Equation (1), Yang et al. proposed another transition method, known as the ‘*Gaussian radial basis function (GRBF)*’, for more general purposes, yet performs the same transition as shown in Figure 11 [16].

**Figure 12:**
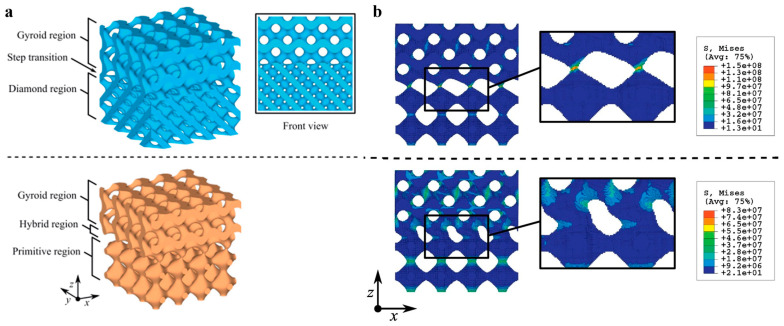
Multi-morphology transition strategy: (**a**) step transition and broad transition (hybrid region) through the sigmoid function of the z parameter and (**b**) von Mises stress at the connecting region of the multi-morphology lattice structure before volume fraction correction steps and after volume fraction correction steps [33].

**Figure 13:**
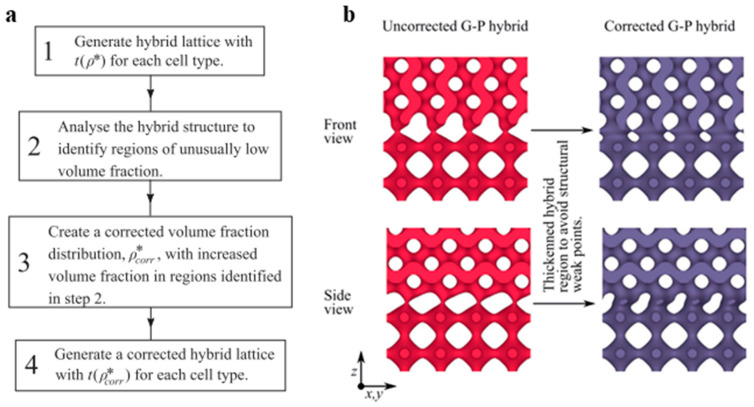
Multi-morphology transition model: (**a**) proposed design approach for multi-morphology lattice structures, and (**b**) uncorrected and corrected hybrid regions [33].

Maskery et al. classified the transition regions into two types: (1) *Step transition*—an abrupt transition between cell types, and (2) *Broad transition*—the transition is gradual and takes place over the length, which gives rise to an intermediate region of unique properties called the ‘hybrid region’ [6]. Figure 12a shows the two types of boundaries generated by the sigmoid function of the ‘z’ parameter [6]. The study illustrated that the abrupt steps in the structures are inhomogeneous, responsible for the failure of structures. On the other hand, a smooth transition through a hybrid region makes load transfer effective and increases the structural properties of the lattice structures [33]. The study proposed the necessary volume fraction correction steps to reduce the stress concentration at the connecting regions. Figure 12b shows the von Mises stress accumulation around the connection before and after the volume fraction correction is adopted. Figure 13 shows the adopted design approach for the construction of multi-morphology lattice structures with an intermediate hybrid region [33].

**Figure 14:**
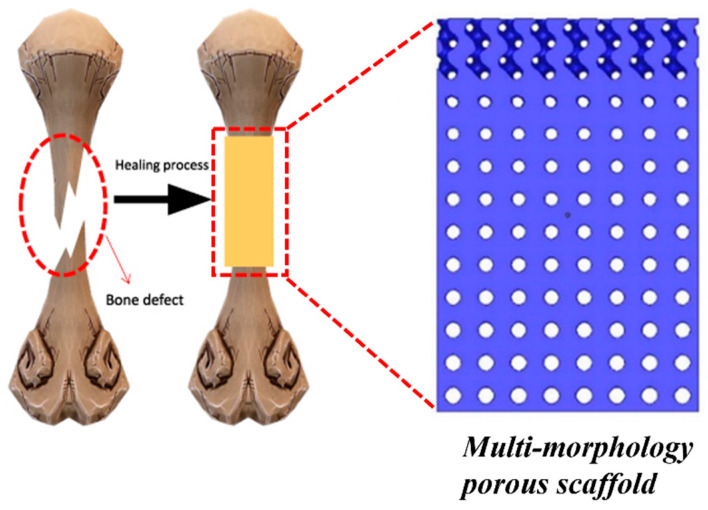
Multi-morphology (Primitive Gyroid)-graded porosity bone scaffold design [15].

Zhu et al. designed TPMS multi-morphology structures with spatially changing pore patterns, as shown in Figure 14. The study concluded that the long-term stability of the implants can be increased by lowering the elastic modulus of the structure. The proposed multi-morphology structure was able to deliver low elastic moduli with high-yield strength [15]. The obtained properties were observed to be useful in strengthening the bone–implant interface and damage-resistant [15].

#### 2.2.2. Truss-Based Multi-Morphology Lattice Structures

**Figure 15:**
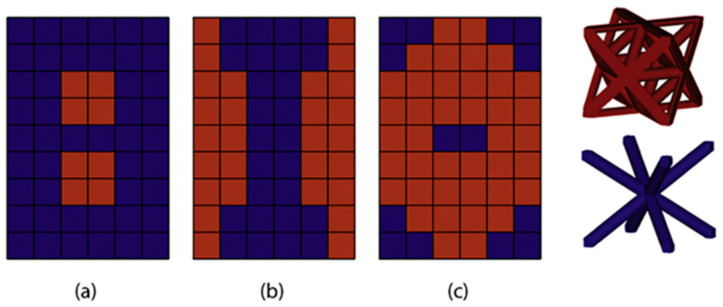
Multi-morphology lattice topologies consisting of BCC and FCC unit cells: (**a**) 25% FCC, (**b**) 50% FCC, and (**c**) 75% FCC [51].

Similar research on multi-morphology structures has also been carried out with traditional truss-based lattice structures. Maxwell number-driven trusses that are classified into bending- and stretch-dominated behaviors are coupled to obtain required functional properties. Bending-dominated structures show characteristic features of lower elastic moduli and flattened plateau stress compared to stretch-dominated structures, which show higher elastic moduli and post-yield softening [8]. The proportion and respective positions of these structures can be altered/adjusted to obtain the required functional properties. Alberdi et al. designed various spatially distributed designs of bending-dominated BCC and stretch-dominated FCC structures, shown in Figure 15, to tune the properties between strength and flexibility [51]. A higher percentage of the BCC truss shown in Figure 15a yields better cushioning behavior due to its low strength and ability to easily deform with a flat plateau region. On the other hand, a higher percentage of the FCC truss, as shown in Figure 15c, yields high-strength mechanical properties.

**Figure 16:**
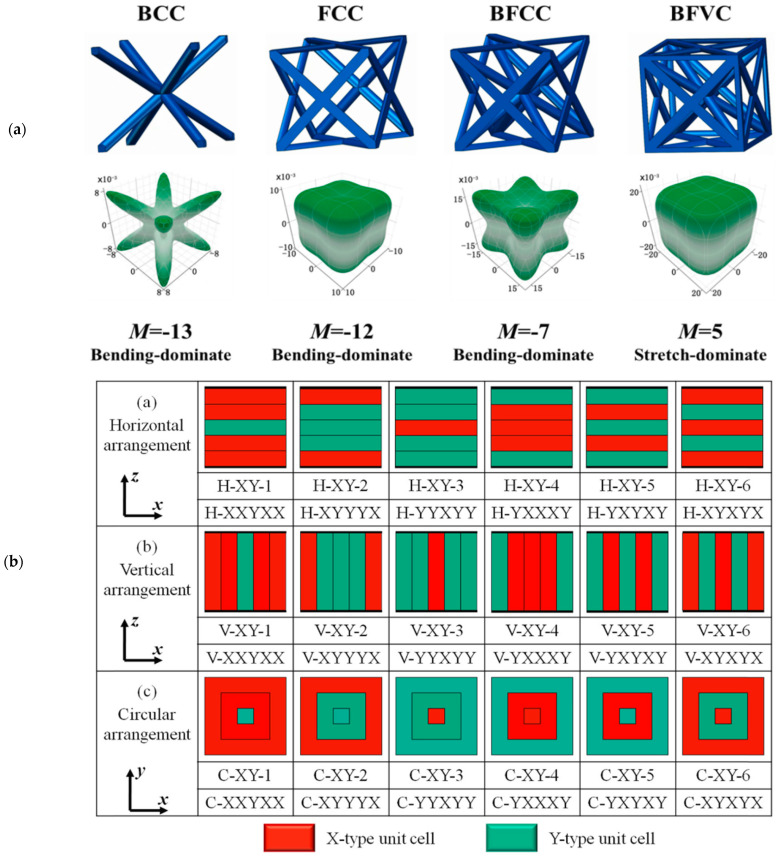
Multi-morphology strut lattices: (**a**) unit cells consisting of different Maxwell numbers, namely BCC, FCC, BFCC, BFVC, and (**b**) horizontal, vertical, and circular arrangements of unit cells [14].

Similar research was carried out by Lei et al. using three bending-dominated structures (BCC, FCC, and BFCC) and one stretch-dominated structure (BFVC), as shown in Figure 16a. Similar to Alberdi et al., Lei et al. used the different spatial distributions of horizontal, vertical, and circular patterns to evaluate structural and functional properties (Figure 16b) [14]. The results obtained show a significant influence of both unit-cell type and its spatial arrangement on the ultimate strength of the structures. Moreover, the study also concluded that the deformation modes can be inter-switched between layer-by-layer and shear failures by the proper adoption of spatial arrangements. The horizontal arrangements proved effective in impact properties compared to vertical and circular arrangements. However, the strength was found to be inferior to a vertical arrangement [14].

**Figure 17:**
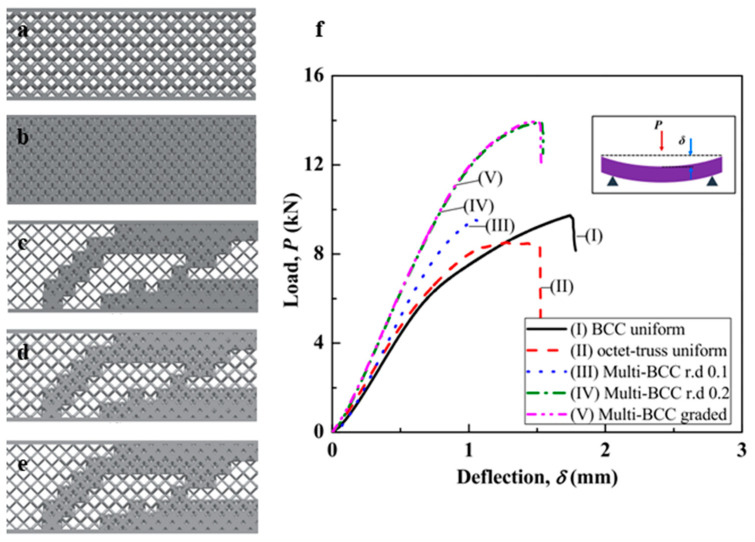
Proposed multi-morphology specimen designs: (**a**) BCC uniform, (**b**) octet-truss uniform, (**c**) multi-BCC with a relative density of 0.1 (BCC—10%; octet—90%), (**d**) multi-BCC with a relative density of 0.2 (BCC—20%; octet—80%), (**e**) multi-BCC graded between relative densities of 0.1 and 0.2, and (**f**) load vs. deflection plots of SLM printed specimens [25].

Kang et al. used a novel topology-optimization strategy to design multi-morphology lattices consisting of BCC (bending-dominated) and octet (stretch-dominated) unit cells. The objective of the study was to evaluate the flexural rigidity of the structures under three-point bending tests [25]. Five different designs were proposed, as shown in Figure 17. These designs were fabricated using the SLM process with optimized printing parameters and carried out a three-point bending test. The load-bearing capacity of the structures, shown in Figure 17, proves the effectiveness of multi-morphology lattice structures over uniform lattice structures [25].

The authors evaluated the overall mechanical performance of all these lattice structures, as listed in Table 1. The results listed in Table 1 show that a multi-morphology lattice structure with a BCC relative density of 20% shows the highest stiffness and maximum load-bearing capacity with appreciable deflection. The structure shows the highest relative flexural rigidity among all the designs, which makes it a more efficient design against bending failures *(Note: relative flexural rigidity is the flexural rigidity of the lattice structure in comparison to the solid of the same dimensions)* [25]. Furthermore, the study also reported that multi-morphology structures perform better in shape maintenance and fracture control than uniform structures, even if excessive deformation or breakage has occurred.

**Figure 18:**
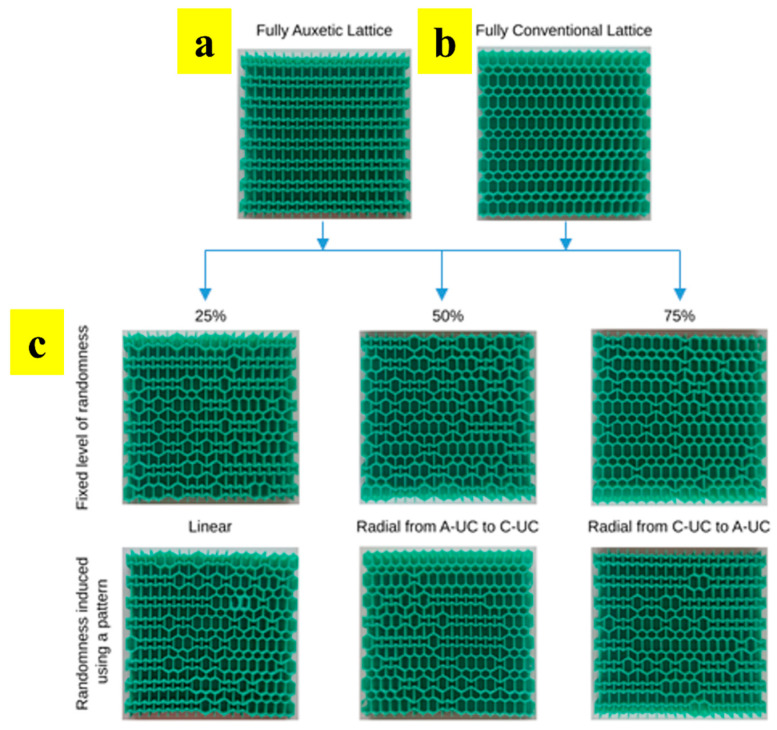
Multi-morphology lattice structures: (**a**) fully auxetic lattice, (**b**) fully conventional lattice, and (**c**) lattice with different % of randomness designed using conventional and auxetic unit cells [29].

Another novel strategy was presented by Mirzaali et al., where conventional unit cells were merged with auxetic unit cells to tailor elastic properties independently (i.e., elastic modulus and Poisson’s ratio) [29]. They merged fully auxetic (Figure 18a) and fully conventional lattices (Figure 18b) into uniquely designed multi-morphology structures that provide a broad range of elastic properties as per the requirement (Figure 18c).

#### 2.2.3. Mixture of Surface- and Truss-Based Multi-Morphology Lattice Structures—Nested Lattice Structures (NLSs)

**Figure 19:**
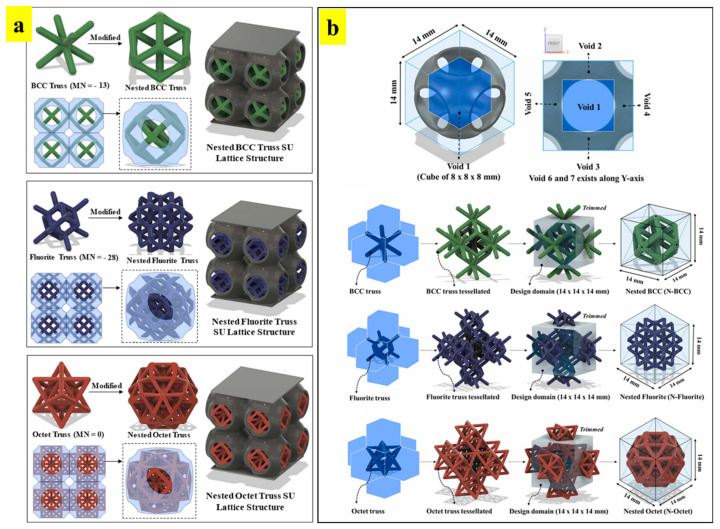
Nested lattice structures: (**a**) different types of nested lattice structures based on BCC, fluorite, and octet trusses, and (**b**) design strategy employed for developing these structures [41].

Bhat et al. introduced a novel concept of fusing different types of trusses into surface lattice structures to deliver multi-functional tunable properties [41]. These lattice structures were named ‘*nested lattice structures (NLSs)*’. These multi-morphology lattices comprise an outer surface-based unit-cell and internal trusses, as shown in Figure 19a. As can be seen in Figure 19a, different types of trusses are used to tune the structural and functional responses. The trusses were categorized into bending-dominated and stretch-dominated, based on the Maxwell stability criterion (i.e., Maxwell number: MN) [8]. The original designs of the trusses were modified based on the void spaces available in the surface-based sea urchin (SU) unit cell, as shown in Figure 19b. From Figure 19b, it can be observed that a total of 7 void spaces exists in the hollow sea urchin unit cell. Based on the void spaces, the trusses were tessellated to fill in these voids. The trusses that protrude out of the design boundary are trimmed off, as shown in Figure 19b.

**Figure 20:**
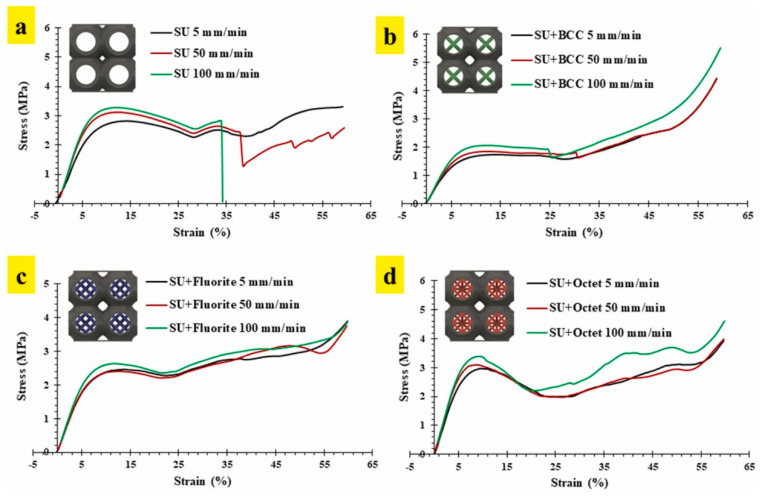
Stress–strain relationship of lattice structures at different static strain rates of 5 mm/min, 50 mm/min, and 100 mm/min: (**a**) empty sea urchin (SU) lattice structure, (**b**) nested lattice structure with BCC truss, (**c**) nested lattice structure with fluorite truss, and (**d**) nested lattice structure with octet truss [41].

The study reported that each nested lattice structure showcased a unique structural and functional response, which was a blend of truss and surface lattice structures. The structures with nested BCC trusses showed a unique flat plateau region with the highest crash force efficiency of 95.79%. On the other hand, the structure with an octet truss demonstrated the highest elastic modulus (52.5 MPa), peak compressive strength (3 MPa), and energy absorption capacity (1360 kJ/m^3^) [41]. The study also reported that the use of internal trusses improved the stability of the structures under random compressive loads. Figure 20 shows that the hollow sea urchin unit cell fails to deliver the same response at higher static strain loads of 50 and 100 mm/min. However, with the use of internal trusses, uniform structural responses can be obtained at different static strain rates of 5, 50, and 100 mm/min.

**Figure 21:**
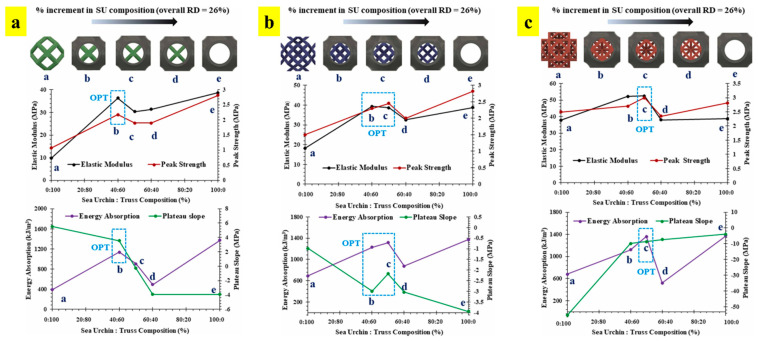
Variation in structural and functional properties with mutual changes in the composition of sea urchin (SU) surface and truss in nested lattice structures: (**a**) BCC, (**b**) fluorite, and (**c**) octet (OPT: optimized composition) [41].

The study further evaluated the mutual percentage of the truss and surface to obtain optimized structural and functional properties [41]. Figure 21 shows the variation in properties with a change in the mutual composition of surface and truss relative densities (i.e., a–e wherein ‘a’ represents 100% strut unit and ‘e’ represents 100% sea urchin unit). The optimized percentage is marked by the composition at which each structure delivers the highest properties, as shown in Figure 21. Thus, with the careful selection of trusses and their relative densities, tunable structural and functional properties can be obtained.

### 2.3. Unit-Cell Tessellation-Based Lattice Structures

Some of the researchers have successfully framed the strategies of varying the spatial arrangement of unit cells to obtain different structural and functional properties. This strategy of filling up the design domain with various spatial arrangements to obtain different responses is termed ‘Tessellation’ [12,55,56,57,58]. Tessellations can be seen throughout nature based on the different adaptation requirements [59]. Such tessellations can be understood and replicated in the form of cellular structures to obtain multi-functional properties. Bhate et al. emphasized the importance of tessellation in cellular structures and classified different ways of achieving it. Most of the cellular structures follow periodic tessellation, where a unit cell is spatially distributed to fill the design domain [59]. Periodic tessellation is further classified into edge-to-edge (ETE), non-edge-to-edge (NETE), and overlapping types, depending on the connectivity between two adjacent unit cells [59]. Most of the lattice structures designed by the researchers have edge-to-edge tessellation, where two unit cells share a common edge/surface completely [56,57]. This complete sharing ensures the proper transfer of the load from one layer of the lattice structure to the next successive layer [57]. Several naturally occurring edge-to-edge tessellations can be traced in nature, where creatures have adapted such kinds of arrangements to fulfill the functional requirements/adaptations. The most common and popular example is that of the honeybee nest, where a hexagonal prism ensures better stability, strength, and storage capacity [59,60,61,62,63,64]. A similar kind of arrangement can also be seen in the endoskeletons of ray fish and abalone shells that facilitate high bending flexibility and excellent compressive strength [65,66,67,68,69]. The arrangement of hexagonal patterns in radiolarian shells provides high strength under hydrostatic pressure [70,71]. Unlike edge-to-edge tessellations, non-edge-to-edge tessellations follow partial edge/surface sharing with adjacent unit cells [12]. The strategy behind this type of tessellation is to manipulate the path of load transfer as per the requirement. Such manipulation could ensure better fail-safe design conditions [57]. This unique form of tessellation draws inspiration from the arrangement of atoms and molecules in crystal structures [12].

**Figure 22:**
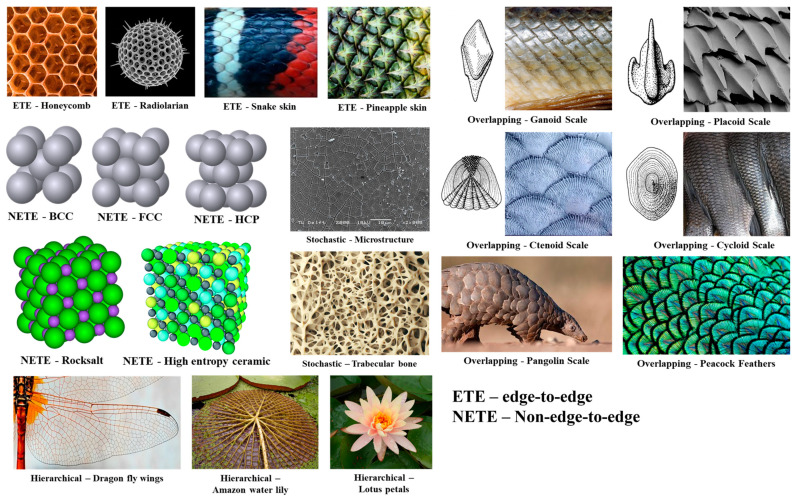
Various types of naturally occurring tessellations in nature based on the adaptation.

One more unique method of tessellation is by overlapping one unit cell over the other. This type of tessellation is known as ‘overlapping tessellation’ [58]. Several oceanic creatures exploit this type of tessellation for mobility. The tessellation strategy, along with unique surfaces, helps in reducing drag force, thereby increasing their speed of swimming. Figure 22 shows four different types of scales in aquatic creatures that follow overlapping tessellation patterns. Similar overlapping tessellations are also seen in pangolins, which helps with protection without compromising their flexibility to bend. The feathers of a peacock, which are extremely big compared to the size of the body of the peacock, follow overlapping tessellations to confine its space. The other class of naturally occurring tessellation is hierarchical tessellation, which includes naturally occurring examples of the wings of a dragonfly, veins in plant leaves, flower petals, etc. [59]. Stochastic tessellation is another class of tessellation that includes a random arrangement of interconnected truss elements. The trabecular bone is the best example of stochastic tessellation, which has an excellent strength-to-weight ratio. The micro-structure of crystals also can be quoted as an example of this class of tessellation [59]. Figure 22 shows various nature-inspired tessellations for adaptive structural and functional properties.

**Figure 23:**
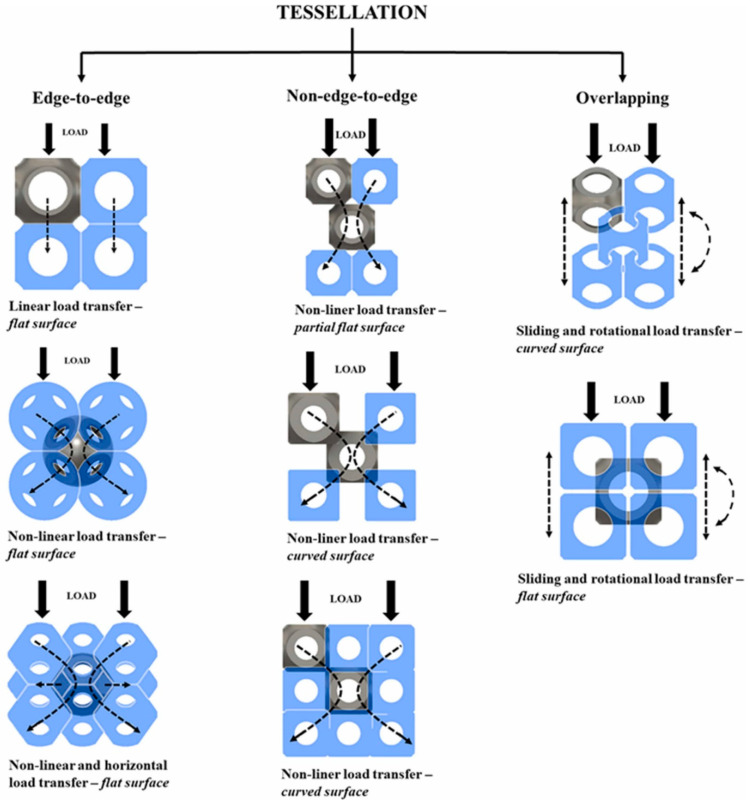
Load transfer paths in different types of tessellations [57].

Bhat et al. attempted to pictorially showcase the importance of different types of tessellations in generating different responses in lattice structures [57]. Based on the stimulus, different responses can be designed by carefully manipulating spatial arrangements (i.e., tessellations). Though load is considered as the stimulus in Figure 23, the concept can be extended to different stimuli, such as fluid and thermal flow, vibration, etc. Figure 23 shows that different tessellated lattice structures transfer the load from the top to the bottom in different patterns. Such a manipulation of the load transfer mechanism/path generates different structural and functional responses in the structures using the same material. Furthermore, it can also be observed in Figure 23 that the relative motion between the unit cells can be obtained by following overlapping tessellation [57].

#### 2.3.1. Non-Edge-to-Edge (NETE) Tessellated Lattice Structures

**Figure 24:**
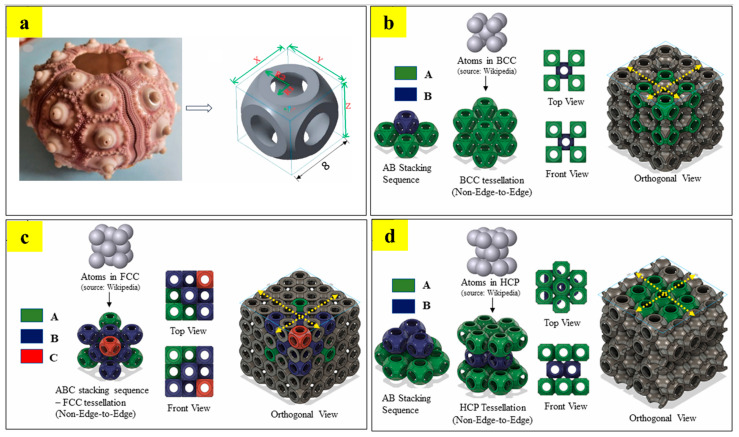
Non-edge-to-edge design strategies inspired by cubic metallic crystal structures: (**a**) bio-inspired sea urchin unit cell, (**b**) BCC tessellation, (**c**) FCC tessellation, and (**d**) HCP tessellation [12].

The design strategy of non-edge-to-edge tessellation (NETE) involves partial edge/surface sharing between adjacent unit cells, as shown in Figure 24. Such a spatial arrangement of unit cells induces a zig-zag load transfer direction, which generates a range of structural and functional properties using a single material [57]. Bhat et al. proposed several different NETE design strategies for tessellating a bio-inspired sea urchin unit cell [12]. The unit cells were tessellated as per the atomic arrangements in the cubic metallic crystal structures (i.e., BCC, FCC, and HCP). These designed structures were termed as ‘*atomic tessellations*’ [12]. All the tessellated lattice structures are confined to the uniform design domain of 32 ± 5 mm. Figure 24a shows the design of a bio-inspired sea urchin unit cell. Figure 24b–d show the different stacking strategies to mimic the cubic metallic crystal structures of BCC, FCC, and HCP, respectively [12].

**Figure 25:**
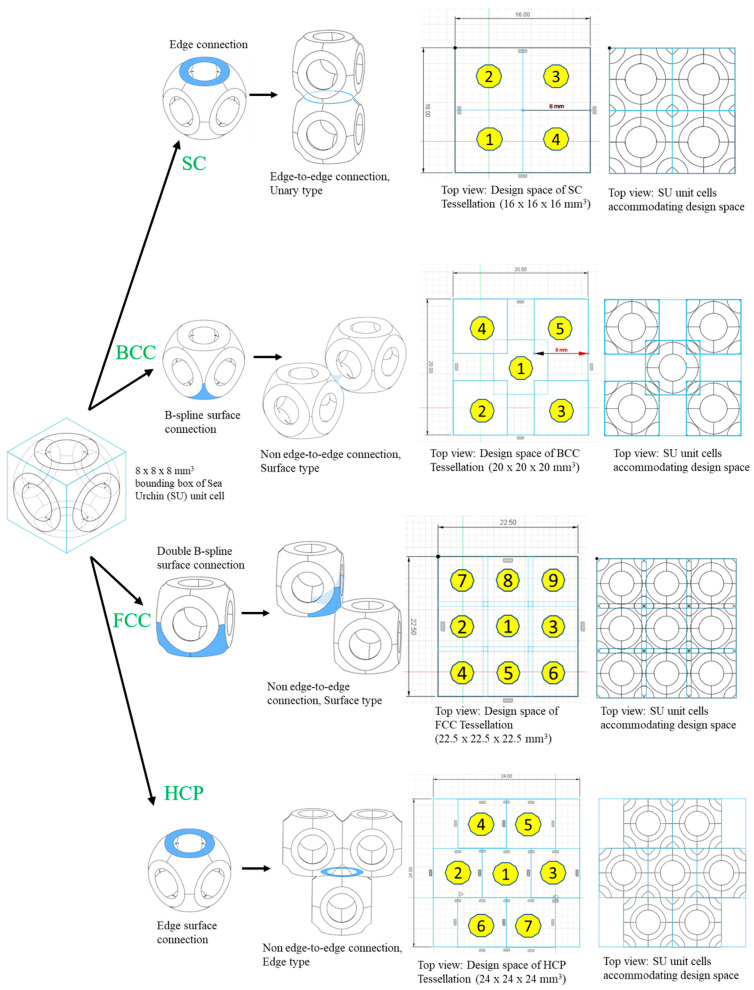
Different types of non-edge-to-edge tessellations (BCC, FCC, and HCP) in comparison with the edge-to-edge tessellation (SC) [Note: the numbers represent relative positions of the unit cells] [12].

Furthermore, the study also highlights the different ways in which non-edge-to-edge tessellations can be achieved. Unlike the SC tessellations in Figure 25, BCC, FCC, and HCP tessellations show unique types of non-edge-to-edge tessellations. BCC tessellation is formed by the partial sharing of spline surfaces of the adjacent unit cells and propagating them across the design domain, as shown in Figure 25. A similar strategy is also adopted in the design of FCC tessellation, with slightly differences in the plane of unit cells. In Figure 25, it can be observed that the adjacent unit cells of BCC tessellations are not in the same plane, whereas in FCC tessellation, all the adjacent unit cells are in the same plane. Contrary to BCC and FCC tessellations, HCP tessellation is formed by the partial sharing of edges of adjacent unit cells (Figure 25) [12].

**Figure 26:**
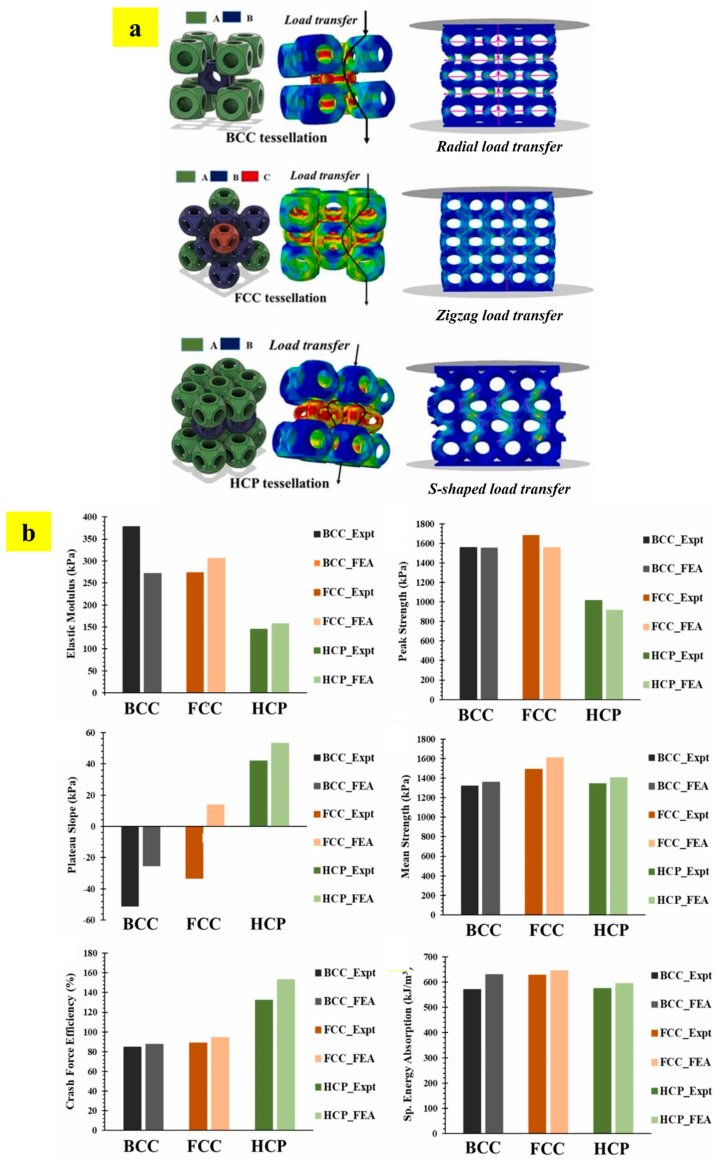
Experimental and numerical analysis of designed NETE tessellated lattice structures: (**a**) different load transfer paths and (**b**) structural and functional properties [57].

The designed NETE tessellated lattice structures were fabricated with HP multi-jet powder bed fusion technology and we evaluated their structural and functional properties using experimental and numerical analyses. The study reports three different types of load transfers, as shown in Figure 26a [57]. These different load transfer mechanisms deliver different properties, such as elastic modulus, peak strength, plateau slope, mean strength, crash force efficiency, and specific energy absorption (Figure 26b) [57]. It can be observed in Figure 26b that BCC tessellated lattice structures exhibit an excellent elastic modulus, while the FCC tessellated lattice structure shows the highest peak strength and energy absorption capacity. Among the three tessellations, HCP tessellation demonstrates the unique feature of a positive slope in the plateau region, which is the characteristic feature of bending-dominated structures [12,57]. Unlike the stretch-dominated behavior demonstrated by BCC and FCC tessellations, bending-dominated behavior delivers better fail-safe design criteria. This is reflected in terms of the crash force efficiency (CFE), where the HCP structure shows > 100% CFE [57]. Thus, the study has successfully demonstrated the role of tessellation in delivering multi-functional properties [12,57].

**Figure 27:**
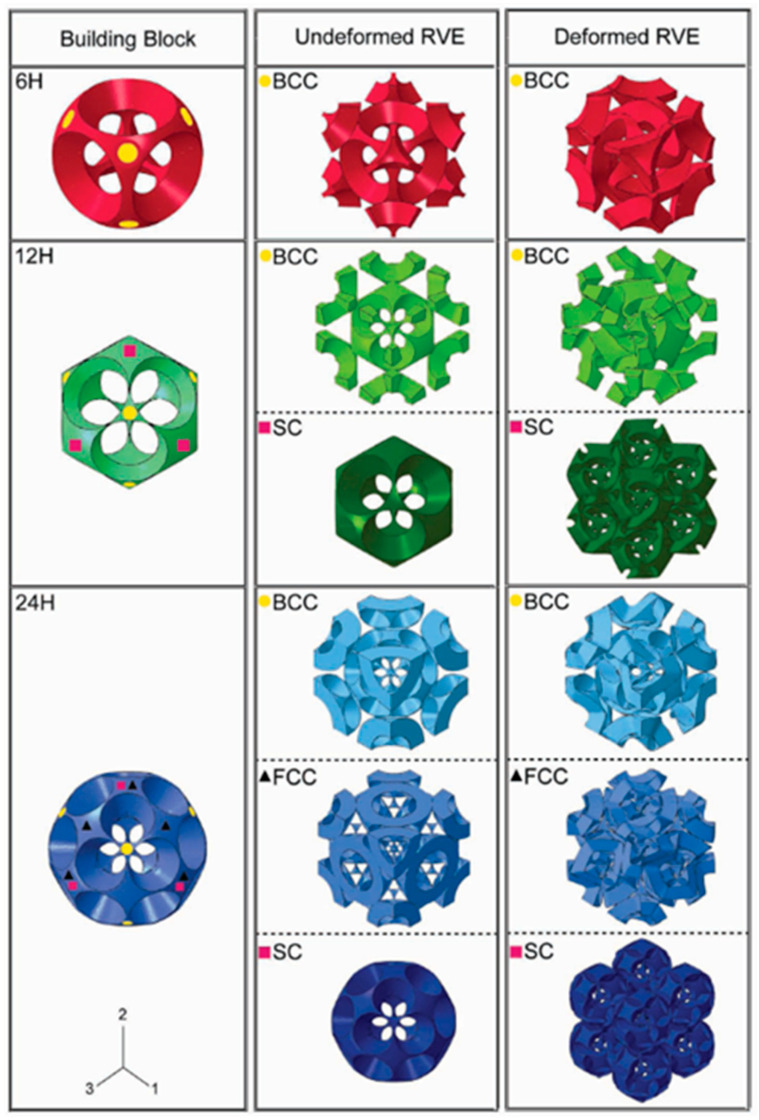
Cubic crystal-inspired spatial arrangements of unit cells (bucklicrystal unit cells) with a prescribed number of holes [72].

**Figure 28:**
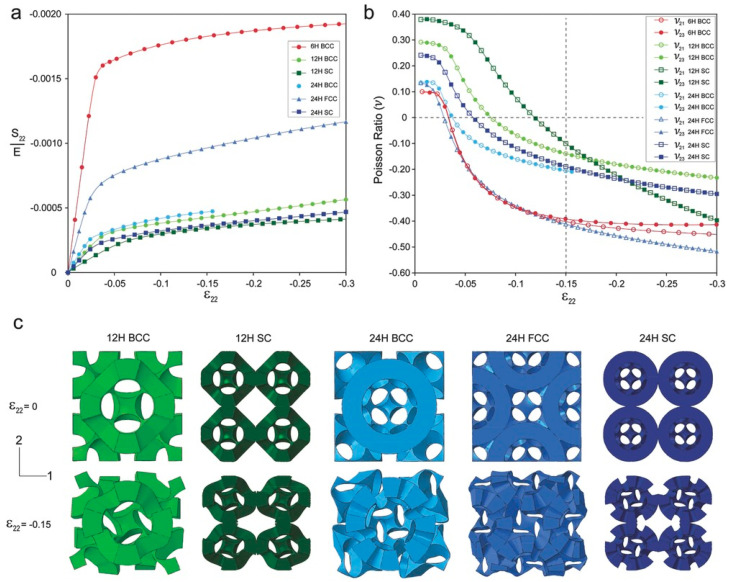
(**a**) Nominal stress–strain curves for various spatially arranged structures, (**b**) all the spatially arranged structures showing a negative Poisson’s ratio, and (**c**) deformation mode of all the structures [72].

A similar study was also reported by Babaee et al. proposing the design of *‘bucklicrystals’* in generating auxetic behavior upon compression. These lattice structures were also inspired by cubic crystal structure arrangements. The developed structures had potential application in buckling-dominated areas due to their high energy absorption capacity. Figure 27 shows various designed bucklicrystals, inspired by simple cubic (SC), body-centered cubic (BCC), and face-centered cubic (FCC) arrangements. A spherical unit cell is created with three different number of holes, i.e., 6 holes, 12 holes, and 24 holes. Figure 28 shows the positions where the immature unit cells can be placed to create different structures of SC, BCC, and FCC out of a single-unit cell. The structures were fabricated with silicon-based rubber with a Young’s modulus of 784 KPa. The size of each unit cell was maintained to be 19.8 mm, with a 7.1 mm wall thickness. The deformation mechanism along with nominal stress–strain behavior are depicted in Figure 28. Figure 28 also shows the auxetic behavior, where the Poisson’s ratio turns negative with an increased strain in the loading direction [72].

**Figure 29:**
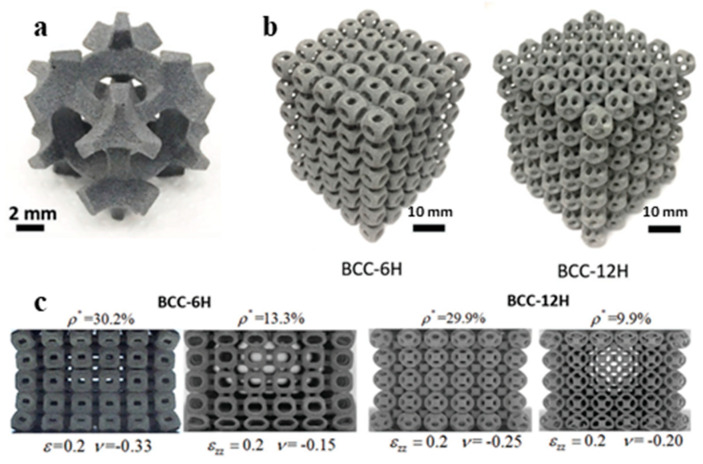
(**a**) BCC 6-hole unit cell with its adjacent children cells, printed using PA-12 material; (**b**) lattice structures: BCC—6H and BCC—12H; and (**c**) auxetic behavior of BCC—6H and BCC—12H structures upon compression [73].

**Figure 30:**
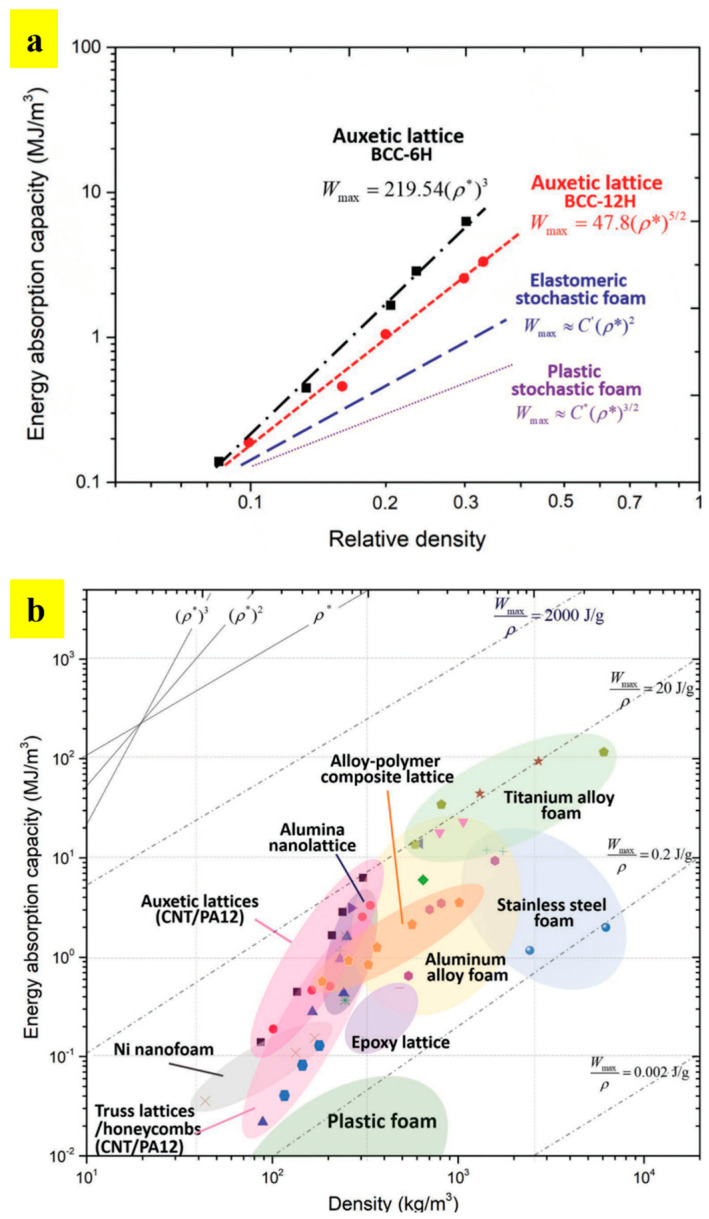
(**a**) Energy absorption capacity comparison of BCC structures with elastomeric and plastic stochastic foams and (**b**) comparison of auxetic lattice structure energy absorption with different materials of different densities [73].

Yuan et al. printed these structures using PA-12 and TPU material through SLS, and reported that the energy absorption capacity is exponentially scaled with an increase in the relative density [73,74]. Figure 29 shows the BCC 6-hole unit cell, lattice structures, and the auxetic behavior of BCC 6-hole and BCC 12-hole lattice structures obtained during the compression test. The study reports that the energy absorption capacity of these spatially arranged structures are exceptionally good with an increasing relative density. Figure 30a shows the energy absorption capacities of BCC—6H and BCC—12H structures being compared with the energy absorption capacities of elastomeric stochastic foam and plastic stochastic foam. The efficiency of energy absorption was observed to be increasing at an increased relative density [73]. These newly designed structures are good for energy absorption among their respective counterparts available in the market. Figure 30b shows that an acceptable range of energy absorption can be achieved with an extremely low density. Thus, these designed structures find use in a number of industrial and packaging industries, where energy absorption and lightweight are the two important parameters [73].

#### 2.3.2. Edge-to-Edge (ETE) Tessellated Lattice Structures

**Figure 31:**
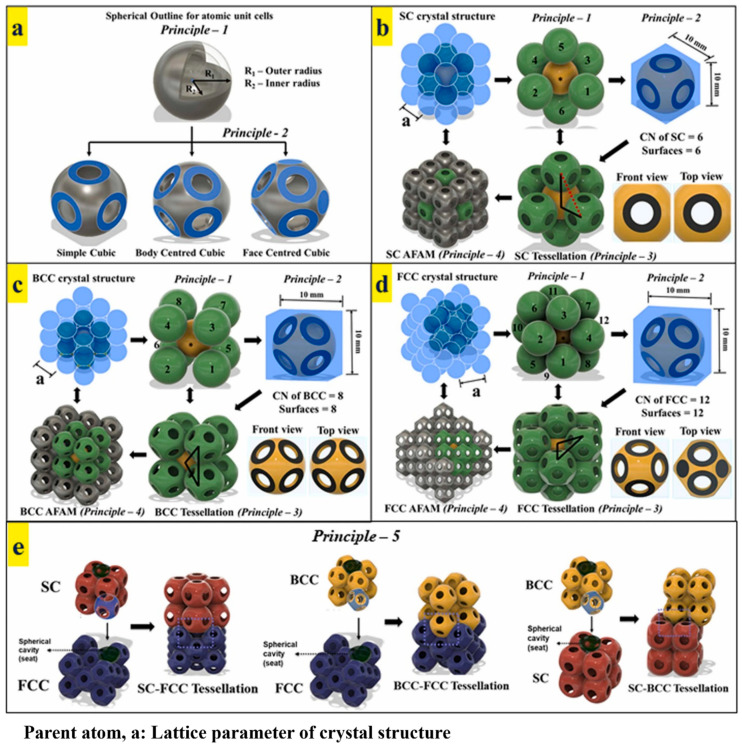
Cubic metallic crystal structure-inspired design of edge-to-edge tessellated materials: (**a**) unit cells of SC, BCC, and FCC tessellations derived from a spherical shell outline, (**b**) design of SC tessellation, (**c**) design of BCC tessellation, (**d**) design of FCC tessellation, and (**e**) interlocking of SC-FCC, BCC-FCC, and SC-BCC tessellations [56].

The motivation behind the design and development of edge-to-edge tessellations is to deliver extremely high structural and functional performances. Complete edge/surface sharing between the adjacent unit cells delivers excellent stability against the load. Based on the cubic metallic crystal structures, Bhat et al. designed three different tessellations, namely: SC, BCC, and HCP. The designs of these lattice structures are framed by formulating closely monitored natural principles [56]. These principles guide designers to develop high-performance lattice structures. Figure 31 shows the design principles employed for developing edge-to-edge tessellated lattice structures. Figure 31a shows the basic spherical structure, which is commonly found in nature due to its least energy state. Such an energy level delivers excellent stability against loads and other stimuli. Based on the coordination numbers of SC, BCC, and FCC, the number of surfaces is generated on the spherical unit cell, as shown in Figure 31a. The adjacent unit cells are stacked according to the coordination number of each tessellation, as shown in Figure 31b (SC), Figure 31c (BCC), and Figure 31d (FCC). It is worth mentioning that the rule of triangulation plays a critical role in generating functional properties. The rule of triangulation states that structures connected in the form of an equilateral triangle deliver excellent stability against load and, thus, are good for load-bearing applications. On the other hand, the structures that violate the rule of triangulation are highly unstable and, therefore, good for cushioning applications [56,61]. In Figure 31b, it can be observed that the SC tessellation violates the rule of triangulation, due to which the tessellation lacks structural stability. Such a tessellation is beneficial for generating cushioning behavior. On the other hand, BCC and FCC tessellations in Figure 31c,d, respectively, follow the rule of triangulation, due to which they are used for high-strength load-bearing applications. All the tessellations are replicated in the design domain to generate SC, BCC, and FCC tessellated lattice structures, as shown in Figure 31b–d, respectively. Furthermore, due to the similar precursor design of each unit cell (i.e., spherical), these tessellations can be stacked onto and interlocked with each other, as shown in Figure 31e. Such a mechanism would help in delivering multi-functional properties using a single material [56].

**Figure 32:**
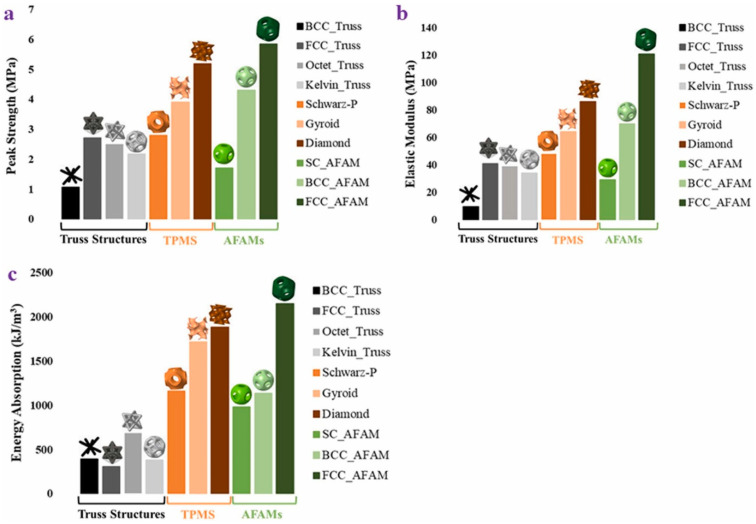
Comparison of properties of edge-to-edge tessellated lattice structures with truss- and surface-based structures: (**a**) compressive peak strength, (**b**) elastic modulus, and (**c**) specific energy absorption capacity [56].

The study reported the use of HP multi-jet powder bed fusion technology to fabricate all the designed samples. Quasi-static compression tests were carried out to evaluate the structural and functional properties. The study reported that the FCC tessellated lattice structure showed excellent load-bearing properties (i.e., elastic modulus and compressive peak strength) among all the existing truss and surface-based lattice structures (Figure 32). Such a high-strength performance was attributed to the adherence to the rule of triangulation. On the contrary, the SC tessellated lattice structure demonstrated the highest densification strain, which accounts for its better cushioning ability [56].

**Figure 33:**
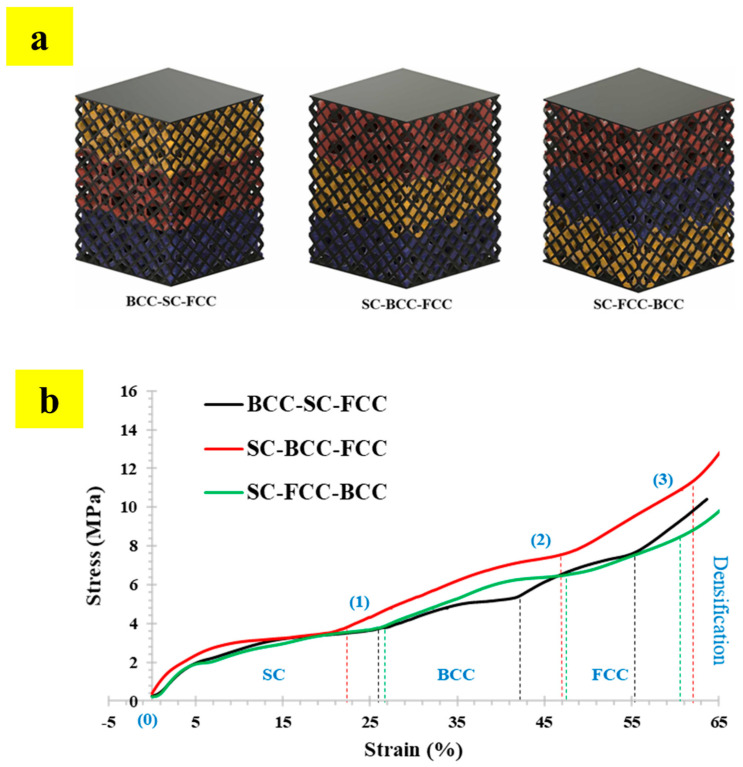
Multi-tessellated lattice structures based on the principle of interlocking: (**a**) different organizations of tessellations and (**b**) their compressive behaviors [56].

The study also reported the use of multi-tessellated lattice structures for generating multi-functional properties, as shown in Figure 33 [56]. Based on the requirements, the proportion of each tessellation in Figure 33a can be carefully adjusted to obtain tunable structural and functional properties. Figure 33b shows the stress vs. strain response of multi-tessellated lattice structures, where the stepped increments can be observed owing to the deformational transition from one tessellation to the other [56]. The transition in deformation behavior can be traced at (1), (2), and (3) of Figure 33b.

#### 2.3.3. Overlapping Tessellation Lattice Structures

**Figure 34:**
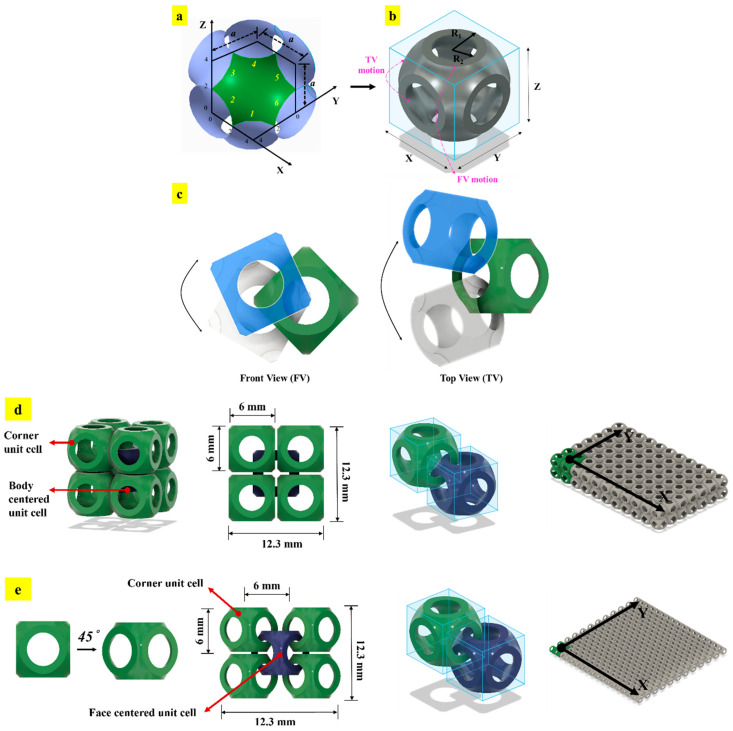
Design strategy of sea urchin (SU) unit cell: (**a**) generated primitive patch with a dimension of *a* × *a* × *a*; (**b**) generated unit cell with outer radius, R_1_, and inner radius, R_2_; (**c**) permitted free movement of interlocked unit cells along up–down (in front view) and left–right (in top view) directions; (**d**) overlapping BCC tessellation strategy and BCC fabric; and (**e**) overlapping FCC tessellation strategy and FCC fabric [58].

Overlapping tessellations are defined as tessellations in which the design domain of each unit cell overlaps with the design domain of an adjacent unit cell [58]. The concept of overlapping tessellation is inspired by the design of the Armadillo carapace, which has excellent flexibility and protection abilities [75,76]. Bhat et al. designed chainmail fabric based on overlapping tessellation strategies [58]. The strategies were designed by utilizing precursor non-edge-to-edge (NETE) tessellations [12]. Similar to non-edge-to-edge (NETE) and edge-to-edge (ETE) tessellations, overlapping tessellations are also inspired by the atomic arrangement of cubic crystal structures (i.e., BCC and FCC). Figure 34 shows the detailed design strategy employed for developing two chainmail fabrics (BCC and FCC). Figure 34a shows the surface topology of the sea urchin unit cell that is used in the study. The required thickness is added to the surface, as shown in Figure 34b. Figure 34c shows the free motion of one unit cell over the other without any locking. Figure 34d,e show the strategies with which BCC and FCC chainmail fabrics were designed, respectively [58].

**Figure 35:**
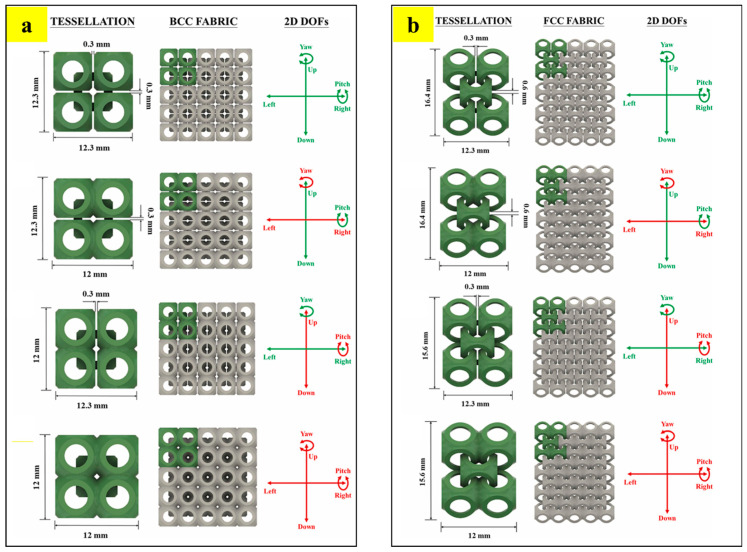
(**a**) BCC-based overlapping tessellation dimensions, generated fabrics, and available degrees of freedom, and (**b**) FCC-based overlapping tessellation dimensions, generated fabrics, and available degrees of freedom (NOTE: green indicates permitted motion and red color arrested motion) [58].

Furthermore, the study also demonstrated the design strategy to selectively arrest the two-dimensional degrees of freedom (2D DOFs). Figure 35a shows different permitted and arrested DOFs of BCC tessellation, where green represents permitted motion and red represents arrested motion. A similar strategy was also employed for FCC fabrics, shown in Figure 35b. With such a strategy, tunable chainmail fabrics capable of delivering multi-functional properties ranging from high strength to high flexibility were developed and demonstrated [58].

All the designed fabrics were fabricated with HP multi-jet powder bed fusion technology and evaluated for their structural and functional properties using experimental tension tests and numerical simulations. The fabrics presented a broad range of properties, ranging from being extremely tough lattice structures to highly flexible chainmail. Such a multi-functional performance can be harnessed for the development of posture-correcting braces, smart wristbands, and protective equipment [58].

## 3. Process Strategies to Obtain Multi-Functional Properties

### 3.1. Multi-Material Additive Manufacturing

**Figure 36:**
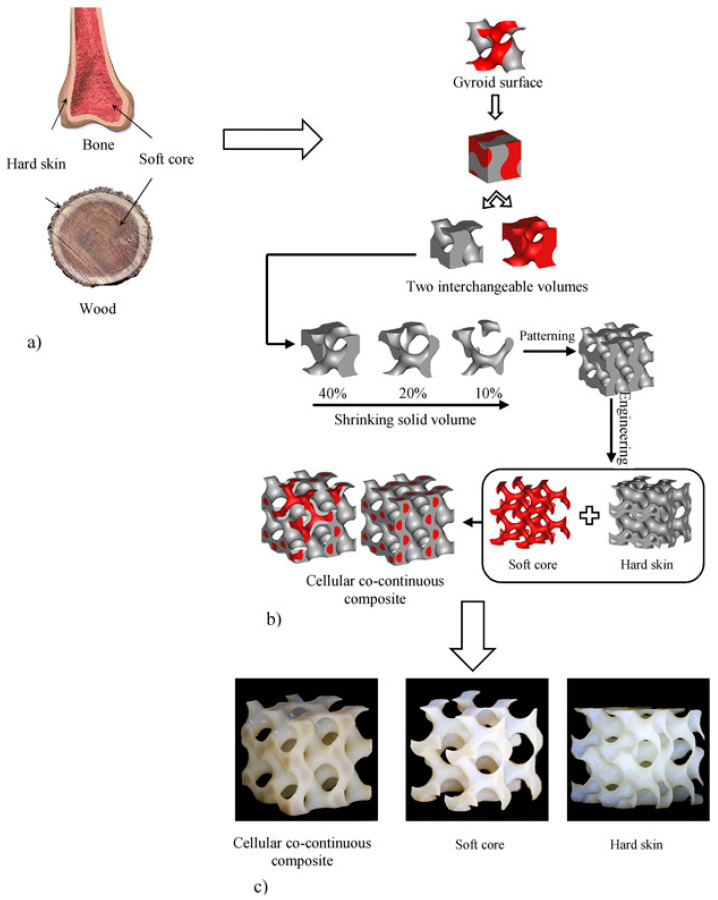
Nature-inspired cellular co-continuous composites: (**a**) natural composites consisting of rigid outer shells and flexible inner cores; (**b**) the process of constructing cellular co-continuous composites inspired by nature begins with the Gyroid surface, which divides the space into two interchangeable domains. The volume may be adjusted and the structure can be patterned and modified to achieve a shell–core structure (**c**) The process of 3D printing is utilized to transform a computer-aided design (CAD) into a tangible object with three-dimensional characteristics [78].

Nature has fascinated scientists and researchers by its use of limited materials to create multi-functional features. For a long time, many scientists have tried to recreate natural surfaces, structures, tessellations, and even complex architecture. The super hydrophobicity, controlled wettability, and self-cleaning properties of lotus leaves, rice leaves, and duck feathers; adhesive properties of gecko feet; and nanopatterns displaying anti-reflective, optical, and iridescent features, etc., are some of nature’s wonderful examples of multi-functional behaviors. Self-assembly and self-organization are other important features of natural materials, such as bones and tooth enamel. Despite these complex structures, they exhibit many interesting properties, like high mechanical strength, lightweight, and toughness. Also, the fracture toughness of bone is remarkably high when compared to its individual constituent materials (i.e., collagen and hydroxyapatite (HAP)). Fracture behavior and high toughness make it an important material from an engineering perspective, since it could be used as a model to design new, tough composites [77]. Bone is not only a composite material composed of collagen and HAP, but is also a composite system of a hard shell and soft core similar to that of wood. A study on nature-inspired cellular co-continuous composites utilized this fact to fabricate multi-material Gyroid structures with a soft core and hard skin (Figure 36) [78].

Other natural materials, like nacre, barnacle, and abalone, have excellent impact-resistant properties due to the way the materials are arranged at a nanoscopic scale. Their material functions are superior to any of the single material phases present, thus inspiring the manufacturing of hybrid composites with inferior building blocks [79]. Similar functions can be achieved by using multiple materials because it is a well-known fact that the mechanical properties of parent material can be enhanced by the addition of one or more materials, making it a composite structure. The secondary material functions as a reinforcement and creates additional functional properties, like sound and vibration damping, thermal or electrical conductivity, and chemical resistance [80,81,82]. Taking inspiration from nature, different types of synthetic composites, like fiber-reinforced, particle-reinforced nanocomposites, and cellular composites have been manufactured. The traditional way of manufacturing composite structures is a capital-intensive and time-consuming process. With the advancement of additive manufacturing, the use of multi-materials coupled with multi-hierarchical structures gives rise to multi-functional composite structures.

Multi-functional composite structure fabrication using additive manufacturing technologies is a budding research domain, given the advantages it has over traditional manufacturing. Additive manufacturing involves the utilization of multi-material printing, where various materials can be deposited in a specific manner. It also involves the use of a pre-blended composite feedstock that contains several types of fillers [83,84]. The selection of the composite structure typically relies on the characteristics of the printing process. Nevertheless, both methods have the ability to impart unique physicochemical features to the resulting materials [85,86]. 

### 3.2. Types of Multi-Material Structures

#### 3.2.1. Fiber-Reinforced Composites

Fiber-reinforced composites, like carbon fiber-reinforced plastics (CFRPs), have been extensively used for high-performance components due to their excellent mechanical properties and lightweight characteristics. The fundamental challenges of fiber-reinforced composites include consolidating the fiber and polymer matrix, control of fiber orientation, and cost of manufacturing [87]. Most fiber-reinforced composites have been manufactured using a two-stage process: (1) material lay-up and (2) consolidation. For consolidation, pressure needs to be applied over the entire composite structure, which requires expensive equipment, thereby increasing the manufacturing costs [88]. The adaptation of additive manufacturing technologies to fabricate composite materials could create a simple composite manufacturing method with lower production costs and a higher degree of automation. The accurate placement of reinforcements allows the laminated structure of the composite to be optimized in each layer, allowing for an improvement in the mechanical performance and design freedom. Fiber-reinforced composites can be categorized as two types.

(a) Short-fiber-reinforced composite

**Figure 37:**
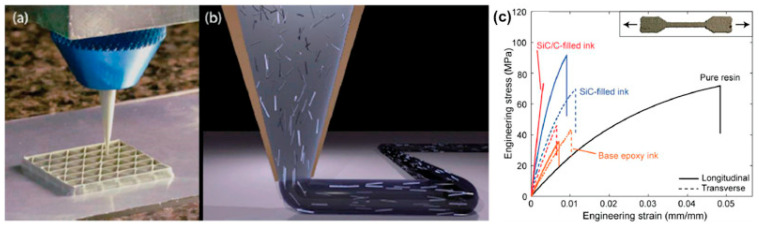
(**a**) An optical image depicting the process of the three-dimensional printing of a composite material in the form of a triangular honeycomb structure. (**b**) Schematic showing the gradual arrangement of elongated fillers with a high aspect ratio inside the nozzle while depositing composite ink. (**c**) The relationship between the applied tensile stress and the resulting strain for 3D-printed tensile bars with different compositions, as well as control samples made from pure epoxy resin.

Short-fiber-reinforced composites have attracted widespread attention due to the ease of manufacturing them at a low cost, along with their superior mechanical properties. Material extrusion processes, such as direct ink writing (DIW) and fused deposition modeling (FDM), have been extensively utilized for manufacturing these composites. For DIW, a viscous liquid of a polymer with fibers homogeneously distributed in it is prepared and extruded, as shown in Figure 37. For FDM, special filaments have to be fabricated by blending thermoplastic polymers and fibers. As for powder-based technologies, making a smooth layer of the powder–fiber mixture is a big challenge [89]. Typical short fibers, including glass fibers [90,91] and carbon fibers (CFs) [92,93,94], are commonly used reinforcements to improve the mechanical properties of polymer composites. The majority of the mechanical properties of the composites are dependent on fiber orientation and void fraction [94]. Additionally, the quantity of fiber content significantly influences the process of short-fiber 3D printing. Currently, the maximum fiber content that may be used for printing is 40 wt.%, and composites with a higher fiber content cannot be printed due to problems with nozzle blockage. Moreover, composites that have a higher concentration of fibers pose challenges to the production of continuous filaments for FDM, mostly due to the decrease in durability. Consequently, the characteristics of the produced composites are constrained by the insufficient amount of fibers present. Gaining a greater comprehension of the rheological characteristics of printing materials and enhancing the fiber content are crucial. Utilizing plasticizers and compatibilizers may enhance the processability of feedstock [90].

##### Continuous Fiber-Reinforced Composite

**Figure 38:**
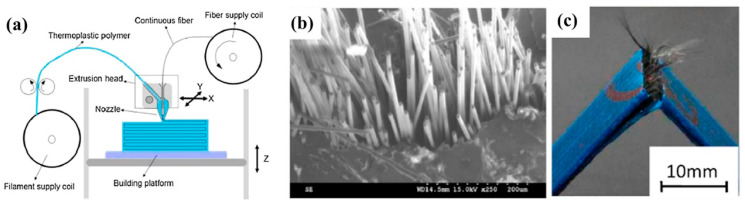
(**a**) The configuration for the additive manufacturing process of continuous fiber-reinforced polymer composites; (**b**) interface micro-structures; (**c**) fracture pattern seen in the cross-section of carbon fiber-reinforced PLA composites.

Multiple studies have documented the use of continuous fiber-based printing, where a continuous fiber is included in the extruded plastic at the nozzle. An investigation was carried out to assess the mechanical characteristics of thermoplastic composites reinforced with continuous fibers, which were produced using a commercially available Mark One printer (Figure 38) [95]. The printed component features a sandwich structure comprising carbon fiber-reinforced thermoplastic (CFRP) in the center, with nylon polymer layers on the top and bottom. Two print heads were used to extrude carbon fiber-reinforced polymer (CFRP) and nylon. Matsuzaki et al. [96] found that the tensile modulus and strength of 3D-printed continuous carbon fiber-reinforced PLA composites are 19.5 (±2.08) GPa and 185.2 (±24.6) MPa, respectively. These values are 599% and 435% higher than the tensile modulus and strength of pure PLA specimens, respectively. The magnitude of this mechanical enhancement is significantly greater when compared to that of short-fiber-reinforced PLA composites. Nevertheless, there are instances where the printed samples still exhibit the irregularity and discontinuity of fibers. While the mechanical characteristics of composites were enhanced to a significant extent, in comparison to pure polymer, the improvement fell short of the theoretical value determined by the law of mixture.

#### 3.2.2. Particle-Reinforced Composites

Particle reinforcements are commonly utilized to enhance the characteristics of a polymer matrix due to their affordability. Particles can be easily blended with polymers, either in the form of a powder for selective laser sintering (SLS), or in a liquid form for stereolithography (SLA). They can also be extruded into printable filaments for the fused deposition modeling (FDM) method. The key issues for consideration when 3D printing particle-reinforced composites include improved tensile strength by adding glass beads [97], iron, or copper particles [98]; improved wear resistance by adding aluminum and aluminum oxide (Al_2_O_3_) [99]; and improved dielectric permittivity by adding ceramic [100] or tungsten [101] particles. In these instances, cuboid or cylinder-shaped components were produced using FDM, SLS, or SLA techniques, resulting in enhanced characteristics. However, no additional structural applications were showcased.

**Figure 39:**
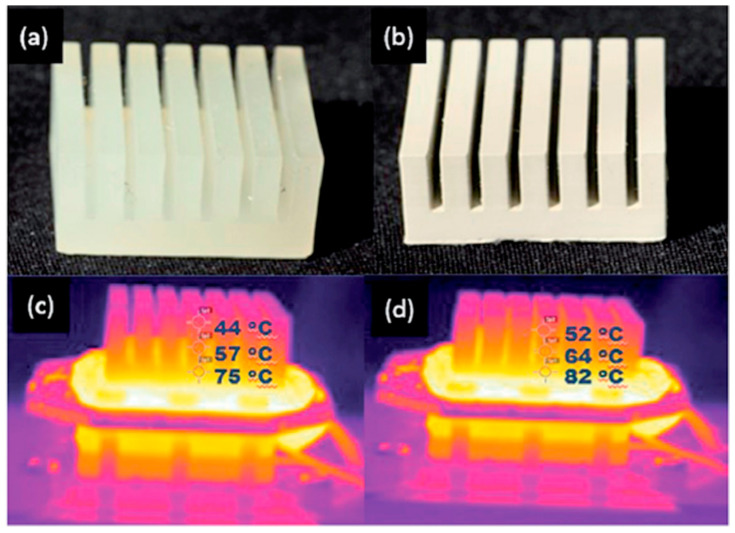
Three-dimensional-printed heat sinks using (**a**) acrylate resin and (**b**) 30% (*w*/*w*) composite material; IR images of (**c**) polymer heat sink and (**d**) composite heat sink heated for 10 min at 100 °C [102].

A noteworthy advancement in the field of 3D printing involves the capacity to manufacture structural components using particle-reinforced composites, which have the potential to be used in practical applications. Kalsoom et al. [102] employed the SLA technique for the additive manufacturing of a heat sink composite structure. This composite construction was composed of micro-diamond particles, which made up to 30% of the total weight of the structure. The particles are embedded in acrylate resins, as seen in Figure 39. The temperature of the composite heat sink was elevated in comparison to that of the pure polymer heat sink, when both sinks were subjected to the same level of heating, hence indicating the enhanced heat transfer rates resulting from the inclusion of diamond particles. In another work, Castles et al. [103] showed the printing of diamond photonic crystal structures using barium titanate (BaTiO_3_)/ABS by FDM. A photonic crystal is a regularly repeating structure composed of materials that have varying dielectric constants. The photonic lattice modifies the physical characteristics, specifically photonic dispersion and scattering. In this study, a combination of two dielectric materials, BaTiO_3_ and ABS, is examined. The addition of BaTiO_3_ particles resulted in enhanced and adaptable dielectric relative permittivity. When BaTiO_3_ loading reached 70 wt.%, the relative permittivity of the printed composite increased by 240% compared to the pure polymer. Furthermore, the dimensional patterning in this study allows for the adjustment of the primary components of the effective permittivity tensor, thanks to the adaptable nature of 3D-printing technology.

Introducing particles into polymers can effectively resolve some challenges encountered during the printing process. The thermal expansion of the polymer causes deformation in the final printed objects, presenting a challenge to the FDM printing process. The efficacy of incorporating metal particles into polymers as a remedy to this issue has been demonstrated [102]. By combining copper and iron particles, ABS composites exhibited a significant decrease in the coefficient of thermal expansion, resulting in a substantial reduction in the distortion of printed objects. An additional attribute of the FDM printing technique is the anisotropic qualities of the 3D-printed component, which can either be advantageous or limiting, depending on the specific application. If the printed item is subjected to isotropic loading conditions, its low tensile strength and modulus in the direction perpendicular to the building orientation may result in the failure of the printed part. The thermoplastic elastomer (TPE) shows potential as an additive for decreasing the anisotropy of mechanical properties. Perez et al. [104] prepared ABS-based composites using TPE, and the results of the tensile test showed a decrease in the disparity between the tensile strength in two perpendicular directions, indicating a reduction in the anisotropy of mechanical properties.

**Figure 40:**
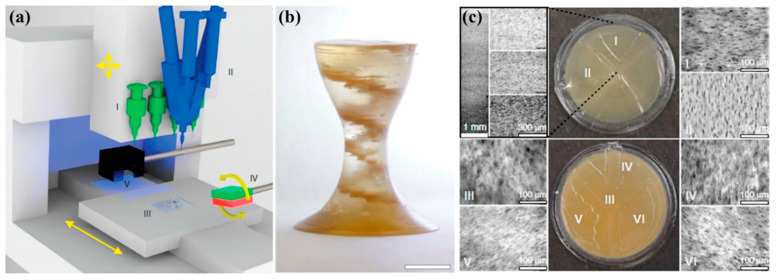
(**a**) Schematics of the magnetically assisted 3D-printing platform for the creation of heterogeneous composites: (I) multiple dispensers, (II) mixing unit, (III) movable head and table, (IV) magnet, and (V) curing unit; (**b**) printed object with an internal helicoidal staircase (scale bar: 5 mm); (**c**) photograph and optical microscope images depicting the structure, emphasizing the successful achievement of the intended gradient in platelet concentration and the localized variation in platelet alignment [105].

In another recent study by Kokkinis et al. [105], a new 3D-printing platform was developed that utilized magnetic assistance. The researchers achieved control over particle orientation by integrating magnetized alumina platelets into a polymer matrix. This can be seen in Figure 40. The increased qualities of printed composite parts in specific orientations are a result of the alignment of anisotropic particles. Additionally, magnetized alumina particles can be mixed with UV-sensitive resins for SLA printing and arranged in a certain direction using magnetic fields when printing [106]. The orientation of magnetized alumina particles was controlled to design and create bio-inspired composite micro-architectures. It was observed that the ensuing mechanical properties of these structures were reliant on their micro-structures.

#### 3.2.3. Nanocomposites

**Figure 41:**
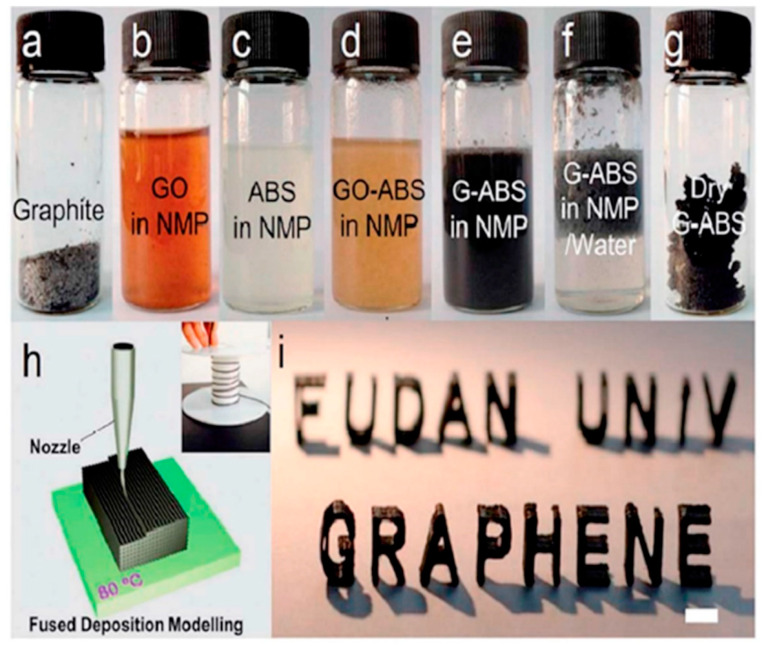
Images depicting (**a**) graphite flakes, (**b**,**c**) suspensions of graphene oxide (GO) and ABS in N-Methylpyrolidone (NMP) solvent, (**d**,**e**) uniform blend of graphene oxide-ABS in N-methyl-2-pyrrolidone (NMP) prior to and following a chemical reduction, (**f**) graphene(G)-ABS coagulations obtained after isolation (**e**) with water, (**g**) G-ABS composite powder after washing and drying, and (**h**) schematic illustration of FFF 3D-printing process. The inset shows graphene-based filament winding on a roller; (**i**) a typical 3D-printed model using a 3.8 wt.% G-ABS composite filament (scale bar: 1 cm) [118].

Nanomaterials, including carbon nanotubes [107], graphene [108], graphite [109], ceramic [110], and metal nanoparticles [111], frequently display distinctive mechanical, electrical, and thermal characteristics. Therefore, incorporating nanoparticles into polymers for printing has the potential to facilitate the production of high-performance functional composites. Nanomaterials have been employed to enhance the mechanical characteristics of printed composite components. The addition of 5 wt.% nano-titanium dioxide (TiO_2_) [104], 10 wt.% carbon nanofiber [112], or 10 wt.% multi-walled carbon nanotube [113] resulted in 13.2%, 39%, and 7.5% enhancements in the tensile strengths of printed composite parts compared to unfilled polymer parts, respectively. However, in all these cases, the printed composite parts exhibited decreased elongation and a more brittle nature. Lin et al. [114] showed that graphene oxide/photopolymer composites produced via selective laser sintering (SLA) exhibit enhanced strength and ductility. Their samples exhibited a significant 62.2% enhancement in tensile strength and a 12.8% improvement in elongation, using only 0.2% graphene oxide (GOs). According to the authors, the enhanced ability of the material to deform without breaking was attributed to the rise in the degree of the crystal structure in the graphene oxide in the strengthened polymers. In addition to improving the mechanical qualities, the incorporation of carbon-based nanomaterials, such as carbon nanotube [115], carbon nanofiber [116], carbon black [117], and graphene [118], can also boost electrical properties. Wei et al. [118] were the first to show that it is possible to use FDM printing to create computer-designed models using a composite of ABS reinforced with graphene. They also found an improvement in the electrical conductivity. By including 5.6 wt.% graphene, the ABS nanocomposites exhibited a significant enhancement in electrical conductivity, with an improvement of four orders of magnitude, as depicted in Figure 41. In addition, the addition of nano-TiO_2_ [119] and nano-clay [120] to the polymer matrix can significantly enhance the thermal stability of printed nanocomposites. In a separate investigation, He et al. [121] created a thermoelectric composite by combining Bi_0.5_Sb_1.5_Te_3_ (BST) with photo resins using the SLA technique. The resulting composites demonstrated a very low thermal conductivity of 0.2 W·m^−1^·K^−1^, making them highly suitable for thermoelectric applications.

A uniform distribution of nanoparticles among polymers is crucial for producing a composite with the necessary properties using the 3D-printing method. In order to prevent the clustering of nanoparticles and promote an effective interaction between nanoparticles and polymers, the chemical surface treatment of nanoparticles was implemented prior to the printing procedures. SLS printers were used to process polystyrene-coated Nano-Al_2_O_3_ particles synthesized using emulsion polymerization [122]. The polystyrene nanocomposites, which were printed using sintering-treated particles, exhibited a compact structure and a significant 300% enhancement in tensile strength. Conversely, samples created with untreated particles displayed minimal increases in their attributes. Another effective method to enhance the interaction between polymers and nanomaterials is by introducing linker molecules that form cross-links with the polymer matrix on the surface of the nanomaterials. After undergoing nitric acid treatment, the oxidized graphite nanoplatelets exhibited greater effectiveness in improving the ultimate strength and Young’s modulus of SLS-printed nylon/graphite nanocomposites [123]. A novel method for generating silver nanoparticles in situ following the printing process was recently proposed. Fantino et al. [124] mixed metal salts with the initial polyethylene glycol diacrylate (PEGDA) liquid photopolymer, and then used a digital-light-processing technique to create 3D structures. The thermal treatment ultimately triggered the in situ formation of metal nanoparticles. The electrical conductivity of silver-reinforced nanocomposites is 1000-times greater than that of pure polymers. This innovative approach partially resolves the challenges associated with incorporating nano-fillers into a polymeric matrix, hence mitigating printing complications.

Three-dimensional printing is a suitable method for producing polymer nanocomposites with functional gradients. It achieves this by distributing varying proportions of nanomaterials to various sections of a building. The capacity to create compositions allows for the maximization of the characteristics of printed components. Chung et al. [125] created a 3D nylon/nanosilica nanocomposite with mechanical properties that change in different regions. They achieved this by employing the SLS process and a 1D nanosilica composition gradient. The printed components demonstrated optimal functional values.

#### 3.2.4. Secondary-Material-Filled Lattice Structures

**Figure 42:**
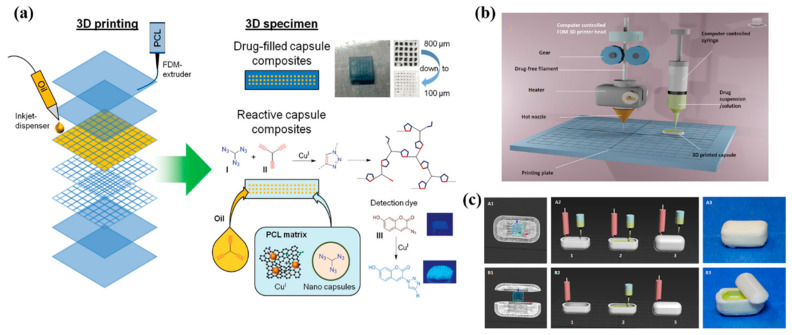
(**a**) Development of the core–shell capsule system by a two-step printing process [126]. (**b**) Schematic illustration of the fabrication of a 3D-printed liquid capsule. A dual-head 3D printer was modified by replacing the right-hand nozzle with a syringe dispenser. (**c**) FDM nozzle and liquid syringe dispenser synchronization occurs in two printing modes: (i) single-phase printing: (A1) the core is in the shell cavity, and (A2) shell printing and capsule filling alternate at each layer. (A3) Completed shell–core designs using a dipyridamole core and eudragit EPO shell. (ii) Multi-phase printing: (B1) the core is situated at a median level between the bottom shell (75%) and the top shell (25%); (B2) the shell is printed first, then the shell bottom is filled; and (B3) the shell top is printed. (B3) Completed shell–core designs (shell top removed from bottom for demonstration) [127].

Multi-material cellular structures are another fascinating concept, where a cellular structure is 3D printed, and the cavities are then impregnated with secondary functional materials to achieve different properties. Filled cellular structures exhibit superior mechanical properties when compared to unfilled structures because of the hydrostatic support presented by the secondary filler material. In a study, an FDM printer is modified in such a way that the primary nozzle prints a core and the second nozzle dispenses liquid inside it, creating capsule-like structures (Figure 42a). These liquid-filled core–shell capsules are 3D printed by interrupting the printing of the shell, subsequently filling the core and then finishing the printing process. The proposed applications in which this can be used are post-printing reactions and damage-sensing applications when combined with appropriate fluids [126].

**Figure 43:**
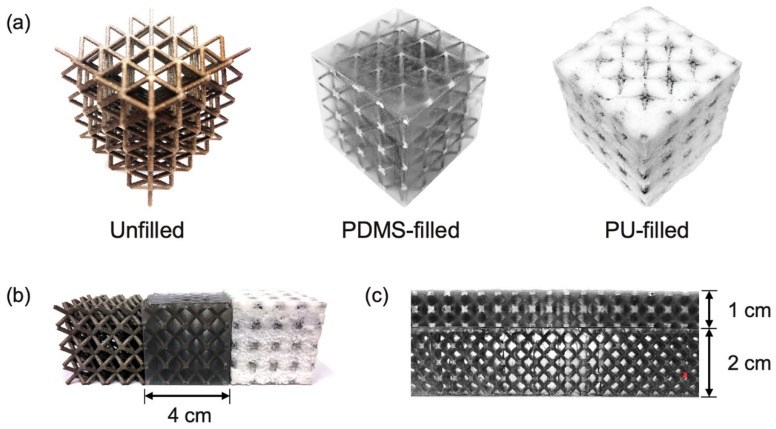
Images of the different types of composite trusses studied. Unfilled, PDMS-filled, and PU-filled trusses (**a**). Examples are shown for trusses studied in (**b**) compression (angled view of three different specimens) and (**c**) bending (top- and side-views of PDMS-filled specimens) conditions [128].

In another study, a similar technique was used to manufacture on-demand patient-specific liquid capsules via coordinated 3D printing and liquid dispensing (Figure 42b,c). This helps healthcare staff to instantly manufacture small doses of liquid capsules with the contents and release pattern as per the patient’s needs. In the domain of lattice structures, Esfahani et al. additively manufactured Ti-6Al-4V trusses and infilled the cavities with several biocompatible thermoplastics. They found that composite trusses infilled with polymers provided enhanced toughness while maintaining stiff structures. This property can be utilized for energy-dissipation applications, such as artificial bone replacement [128]. In another study, BCC trusses additively manufactured with Ti-6Al-4V were filled with an elastomer (poly-dimethyl siloxane (PDMS)) and polyurethane foam (Figure 43). When compared to unfilled trusses, the filled trusses showed improved mechanical properties and delayed compressive failure at higher strains. The authors found an increase in volumetric energy dissipation by 19% and energy absorption efficiency by 12%.

Liquid-filled cellular structures have also been investigated for impact energy absorption. In a study conducted by Soe et al., using FFF process, they 3D printed a hollow conical structure with a thermoplastic elastomer and filled it with water at different infill percentages. They discovered that, for higher impact speeds, water-filled cellular structures showed a better impact absorption capacity when compared to partially liquid-filled and air-filled cellular structures. At lower impact speeds, air-filled cellular structures showed a much better performance when compared to fully-filled structures [129].

Kao et al. 3D-printed cubic lattice structures with polylactic acid (PLA) and filled them with polyurethane foam to investigate the low-velocity impact properties of the structures. They found that flexible foams have better impact properties than rigid foams. Also, they concluded that the foam-filled lattice structures showed a 23% increase in energy absorption when compared to unfilled lattice structures [130]. Cellular structures used for optimum stiffness when combined with reinforcements like foams or liquids filled in closed cells tend to increase the energy absorption property of structures. This makes these structures suitable for items like shoe mid-soles and protective sports gear, like helmets and knee/elbow pads. High-velocity-impact resistance is one of the primary requirements for space equipment. With nearly no resistance to the flow of a projectile in space, even a small projectile can cause great damage to machinery. Using the technology of filled lattice structures to print-on-demand components can greatly improve damage protection.

**Figure 44:**
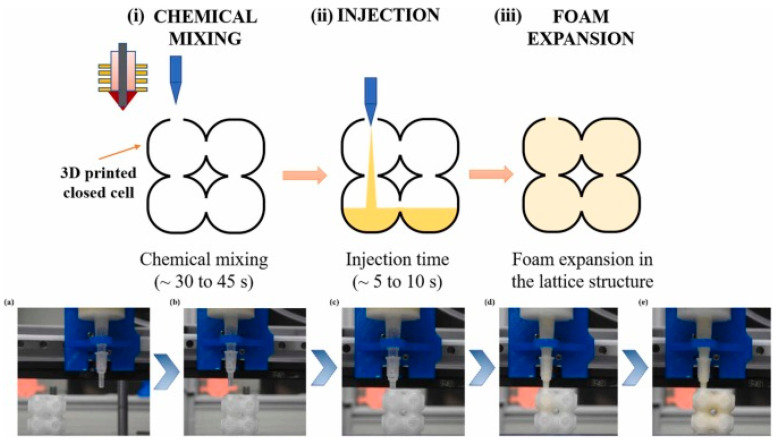
Process of the additive manufacturing of closed-cell lattice structures and PU foam filling with a secondary nozzle [13].

Several studies conducted by Prajapati et al. evaluated the novel concept of AM products composed of PU foam-filled closed-cell lattice structures with a hybrid 3D-printing and foam filling process to achieve lightweight multi-material components with multi-functional properties (Figure 44). The direct digital manufacturing concept is applied to develop an open-source hybrid FFF 3D printer by using a primary extruder for polymer printing and a secondary unit for filling closed cells with PU foam, as per the application and process strategy. Closed SU lattice structures are 3D printed using a TPU filament, and PU foam is dispensed into the lattice structures in a single process [13]. The encapsulation of secondary material in the closed cell can solve interface problems in multi-material AM components. Incompatible materials can also be trapped in closed cells to achieve better mechanical properties. Thus, filled closed cells can be a possible solution for low-cost multi-material AM products for product development [131,132].

**Figure 45:**
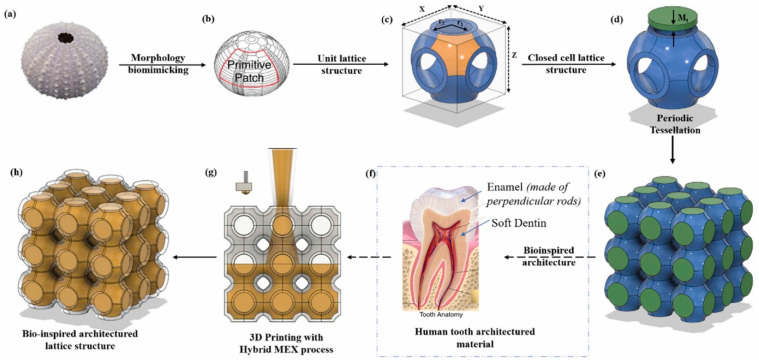
The design evolution of the lattice structure for mechanical anisotropy reduction [133].

Tooth-enamel mechanical anisotropy is remarkably similar to AM products made by the material extrusion (MEX) process (FFF method) (Figure 45). Mechanical anisotropy is the consequence of the layer-wise deposition of MEX-produced components, much like perpendicular rods in teeth. In another aspect of the study, we attempted to reduce mechanical anisotropy by designing a bio-inspired architecture lattice structure using a support-free closed cell. To increase its crashworthiness, PU foam was used as a supplementary material inside the constructions. The PU foam was contained in this closed chamber, which also accommodated the flaws produced during the MEX printing process [133].

## 4. Future Trends and Outlooks for the Multi-Functional Lattice Structures

Multi-functional lattice structures using design and multi-material strategies have shown that their physical properties can be easily manipulated by varying their topology and base materials. The emergence of additive manufacturing has proved to be an effective tool in transforming mass production into mass customization with the development of products possessing multi-functional properties, like lightweight, ultra-stiff/compliant, energy absorbent, and superior toughness [134,135,136]. They can be employed in a wide variety of engineering applications, such as aerospace, automobiles, biomedical implants, and vibration absorption. Custom-made structures can be designed to fit design criteria and developed much faster than the traditional manufacturing process. The freeform design guided by the Design for Additive Manufacturing (DfAM) principles allows a designer to use their imaginative power to turn a concept into reality. The DfAM principles include a set of guidelines that a designer must follow for a particular AM process to achieve the maximum benefit from it. For example, knowledge of the minimum print dimension, support-free overhanging angle, and bridging distance of the FFF process will assist in developing efficient structures that are durable and printed in less time [131].

Inspiration from nature is always appreciated as it focuses on optimum performance with less consumption of material and energy. The recent trends also include the use of artificial intelligence (AI) to generate bio-inspired designs to computationally manipulate mechanical properties using AI-aided designs [137]. Machine learning (ML)-based approaches can become a feasible tool to efficiently predict mechanical properties and save vast computational costs for FEA simulations [137]. By training ML models with the individual tessellation and material combination strategies discussed above, coupled with AM technologies, efficient products with predictable and tunable mechanical properties can be developed with minimum material use and time and energy consumption, leading to green manufacturing. The use of reusable thermoplastics and other bio-sourced materials to develop multi-functional structures will cater to functionality as well as reusability and recyclability. Thus, the incorporation of design and process strategies in AM technologies for product development can become the backbone of future, sustainable, smart manufacturing and Industry 4.0 [32].

## 5. Conclusions

The present review elucidates the state-of-the-art advancements in the field of multi-functional lattice structures fabricated using AM technology. The study successfully categorized the strategies into design and process strategies based on the manipulation of four basic lattice factors (unit-cell morphology, tessellation, relative density, and material characteristics). The design strategies predominantly consist of lattice structures that can be functionally graded using a single-unit-cell morphology (i.e., relative density manipulation), or by combining multiple-unit-cell morphologies, such as truss- and surface-based unit cells (i.e., morphology manipulation). The design strategies also elaborately explained unit-cell tessellation principles, where a single-unit-cell morphology is arranged in different ways in a design space mimicking natural structures to obtain multi-functional properties. The study also highlighted the process strategies explored in the area of multi-material additive manufacturing (i.e., material manipulation) with the use of various composite materials, such as long- and short-fiber-reinforced, particle-reinforced, and nanocomposite structures. It also discusses the new domain of secondary-material-filled lattice structures, where the multi-material aspect can be combined with the lattice structures’ design to achieve multi-functional properties. This study, as a whole, will serve as a guiding light for design enthusiasts to develop function-specific lattice structures by referring to the pool of strategies in this study, in addition to the fact that the study also emphasizes the scope of utilizing various tools, such as DfAM, artificial intelligence, and machine learning, to increase the efficacy and ease of designing multi-functional lattice structures.

## Figures and Tables

**Table 1:** Mechanical performance of five different designs of multi-morphology lattice structures [25].

Design Case	Initial Stiffness (kN/mm)	Max. Load (kN)	Deflection at Max. Load (mm)	Relative Flexural Rigidity
Uniform BCC	9.71	9.74	1.74	0.24
Uniform octet	10.18	8.25	1.15	0.25
Multi-morphology truss (BCC—10%; octet—90%)	12.81	9.41	0.98	0.32
Multi-morphology truss (BCC—20%; octet—80%)	14.18	14.17	1.54	0.35
Multi-diameter BCC truss	13.96	13.85	1.48	0.35

## Data Availability

The data will be made available upon request form the corresponding authors.
